# Novel mechanism of hydrogen peroxide for promoting efficient natamycin synthesis in *Streptomyces*

**DOI:** 10.1128/spectrum.00879-23

**Published:** 2023-09-11

**Authors:** Gongli Zong, Guangxiang Cao, Jiafang Fu, Peipei Zhang, Xi Chen, Wenxiu Yan, Lulu Xin, Zhongxue Wang, Yan Xu, Rongzhen Zhang

**Affiliations:** 1 Key Laboratory of Industrial Biotechnology of Ministry of Education & School of Biotechnology, Jiangnan University, Wuxi, China; 2 Biomedical Sciences College & Shandong Medicinal Biotechnology Centre, Shandong First Medical University & Shandong Academy of Medical Sciences, Ji’nan, China; South China Sea Institute of Oceanology Chinese Academy of Sciences, Guangzhou, China

**Keywords:** natamycin biosynthesis, cholesterol oxidase SgnE, redox-dependent switch, intracellular H_2_O_2 _concentration

## Abstract

The mechanism of regulation of natamycin biosynthesis by Streptomyces in response to oxidative stress is unclear. Here, we first show cholesterol oxidase SgnE, which catalyzes the formation of H_2_O_2_ from sterols, triggered a series of redox-dependent interactions to stimulate natamycin production in *S. gilvosporeus*. In response to reactive oxygen species, residues Cys212 and Cys221 of the H_2_O_2_-sensing consensus sequence of OxyR were oxidized, resulting in conformational changes in the protein: OxyR extended its DNA-binding domain to interact with four motifs of promoter p*_sgnM_*. This acted as a redox-dependent switch to turn on/off gene transcription of *sgnM*, which encodes a cluster-situated regulator, by controlling the affinity between OxyR and p*_sgnM_*, thus regulating the expression of 12 genes in the natamycin biosynthesis gene cluster. OxyR cooperates with SgnR, another cluster-situated regulator and an upstream regulatory factor of SgnM, synergistically modulated natamycin biosynthesis by masking/unmasking the −35 region of p*_sgnM_* depending on the redox state of OxyR in response to the intracellular H_2_O_2_ concentration.

**IMPORTANCE:**

Cholesterol oxidase SgnE is an indispensable factor, with an unclear mechanism, for natamycin biosynthesis in *Streptomyces*. Oxidative stress has been attributed to the natamycin biosynthesis. Here, we show that SgnE catalyzes the formation of H_2_O_2_ from sterols and triggers a series of redox-dependent interactions to stimulate natamycin production in *S. gilvosporeus*. OxyR, which cooperates with SgnR, acted as a redox-dependent switch to turn on/off gene transcription of *sgnM*, which encodes a cluster-situated regulator, by masking/unmasking its −35 region, to control the natamycin biosynthesis gene cluster. This work provides a novel perspective on the crosstalk between intracellular ROS homeostasis and natamycin biosynthesis. Application of these findings will improve antibiotic yields via control of the intracellular redox pressure in *Streptomyces*.

## INTRODUCTION

*Streptomyces* species are saprophytic soil bacteria that produce a diverse array of specialized metabolites, including half of all known antibiotics (1). *Streptomyces* have had a long evolutionary association with fungi, with fungal cell walls being a key source of nutrition (2, 3), and *Streptomyces* spp. play key roles in the natural recycling of materials from cell walls of fungi and plants (4, 5). Sterols are present in membranes of organisms from unicellular fungi to animals and have versatile functions as structural elements and signaling molecules (6).

Natamycin, produced by *S. natalensis* (7) , *S. gilvosporeus* (8), *S. lydicus* (9), and *S. chattanoogensis* (10), is a 26-membered ring glycosylated tetraene antifungal macrolide (7, 11). Its antifungal activity is dependent on hydrophobic interactions between natamycin, which is a polyene, and sterols in the fungal cell wall (12 – 14). It has become one of the major mold inhibitors used in the food industry (13) and has potential for preventing prostate cancer cell proliferation (15). Natamycin biosynthesis is influenced by two cluster-situated regulators SgnR and SgnM in a hierarchical way. SgnR binds *sgnM* promoter and activates its transcription, and the gene product of the latter activates transcription from eight different promoters of natamycin structural genes directly (14). The natamycin biosynthesis gene cluster of *S. gilvosporeus* F607 comprises 20 genes, including *sgnH* encoding an efflux pump; *sgnT* encoding a hydrophilic pimaricin inducer (PI) factor secretion protein; three genes (*sgnS0*, *sgnS1,* and *sgnS2*) encoding type I modular PKSs; *sgnK* encoding mycosamine transferase; *sgnI* encoding a thioesterase; *sgnJ* encoding GDP-mannose 4,6-dehydratase; *sgnA* encoding an ABC transporter; and *sgnD* encoding a cytochrome P450 monooxygenase.

Natamycin biosynthesis gene clusters contain different cholesterol oxidase genes: *pimE* in *S. natalensis* (7), *sgnE* in *S. gilvosporeus* (16, 17), *slnE* in *S. lydicus* (18), and *scnE* in *S. chattanoogensis* (10). Cholesterol oxidases are mainly flavin adenine dinucleotide (FAD)-dependent enzymes that catalyze the oxidation and isomerization of cholesterol to Δ^4^-ene-3β-ketosteroid and H_2_O_2_ (19). Interestingly, cholesterol oxidase genes are often present in gene clusters related to the synthesis of small polyenes: e.g., *tetrO* in tetramycin synthesis (20), *pteG* and *filG* in filipin synthesis (21), and *rimD* in rimocidin/CE-108 synthesis (22). Functional analysis studies showed that the deletion of *pimE* blocked natamycin biosynthesis in *S. natalensis*, while the addition of extracellular cholesterol oxidase to the culture broth or resting cells of the knockout mutant restored natamycin production (19, 23). Wang et al. found that overexpression of cholesterol oxidase improved natamycin production in *S. gilvosporeus* (24). However, the regulatory mechanism of these cholesterol oxidase genes in natamycin synthesis has rarely been reported (19, 23, 24).

H_2_O_2_ is an important reactive oxygen species (ROS) (19), which mediate the toxicity of oxygen because of their high-chemical reactivity and are also important signal transducers in eukaryotes and prokaryotes (25, 26). Relationships between ROS and secondary metabolism have been detected in the doxorubicin-producer *S. peucetius* (27), the actinorhodin-producer *S. coelicolor* A3(2) (28 – 30), the ε-poly-l-lysine-producer *Streptomyces* sp. AF3-44 (31), the clavulanic acid-producer *S. clavuligerus* (32), the validamycin A-producer *S. hygroscopicus* (32), and the avermectin-producer *S. avermitilis* (33). In *S. natalensi*s ATCC 27448, inactivation of the superoxide dismutase-encoding gene *sodF* decreased natamycin production, while suppression of H_2_O_2_-detoxifying enzymes (alkyl hydroperoxidase and catalase) resulted in natamycin overproduction, suggesting that there was crosstalk between oxidative stress and regulatory networks of secondary metabolism (34). An imbalance of intracellular ROS homeostasis triggered by redox-based regulation was found to modulate natamycin biosynthesis in *S. natalensis* (34). Oxidizing agents, such as H_2_O_2_, were found to be degraded by polyene macrolide antibiotics (35, 36). Thus, an H_2_O_2_-sensing pathway was proposed to trigger natamycin biosynthesis, which ultimately balances ROS homeostasis (37).

Previous studies have shown that the peroxide-sensing transcriptional regulators OxyR, SoxR, PerR, and OhrR are involved in oxidative stress defense responses in bacteria (26). In each case, oxidative modification of the regulator alters its DNA-binding properties. The genome of *S. gilvosporeus* F607 contains a gene-encoding OxyR (GenBank no. NZ_CP020569.1) (17). OxyR has an N-terminal DNA-binding domain (DBD) and a C-terminal regulatory domain (RD) and is a member of the LysR family (38). Several studies demonstrated that OxyR was a switch that modulated the transcription of antioxidant genes dependent on the intracellular oxidizing conditions (26, 39, 40). Reduced OxyR has two redox-sensing cysteine residues that can be oxidized to form a disulfide bond, or overoxidized to the S-sulfonate conformation (Cys–SO_3_H), at specific H_2_O_2_ concentrations (26, 41, 42). In this way, the OxyR tetramer functions as a “three-state redox switch,” whereby its extended conformation and DNA-binding sites change to alter its regulatory function to turn on or off the transcription of its target antioxidant genes (43, 44).

Recently, we found that overexpression of SgnE (cholesterol oxidase) enhanced the production of natamycin in *S. gilvosporeus* (24). Here, *S. gilvosporeus* SgnE catalyzed the production of H_2_O_2_ from sterols from plants, fungi, and vertebrates (ergosterol, stigmasterol, and cholesterol, respectively). The H_2_O_2_ acted as signaling molecule to trigger redox-state-dependent gene transcription, protein expression, DNA binding, and enzyme interactions that regulate natamycin biosynthesis.

## MATERIALS AND METHODS

### Strains and cultures

For analyses of sterols as elicitors, *S. gilvosporeus* F607 was cultured in a seed medium containing 10 g/L glucose, 5 g/L peptone, 3 g/L yeast extract, and 3 g/L malt extract for 24 h. Then, 5% of the seed culture was added to TC medium (2.0 g/L soy peptone, 0.45 g/L yeast extract, 2.0 g/L NaCl, 1.0 g/L MgSO_4_, 60.0 g/L glucose, 0.2 g/L stigmasterol, and 0.045 g/L ergosterol, pH 7.5); TH medium (2.0 g/L soy peptone, 0.45 g/L yeast extract, 2.0 g/L NaCl, 1.0 g/L MgSO_4_, 60.0 g/L glucose, 2.0 g/L stigmasterol, and 0.45 g/L ergosterol, pH 7.5); or TF medium (2.0 g/L soy peptone, 0.45 g/L yeast extract, 2.0 g/L NaCl, 1.0 g/L MgSO_4_, 60.0 g/L glucose, 4.0 g/L stigmasterol, and 0.9 g/L ergosterol, pH 7.5). *S. gilvosporeus* F607 was used as the *sgnE* and *oxyR* gene donor. It was routinely cultured in seed medium containing 10 g/L glucose, 5 g/L peptone, 3 g/L yeast extract, and 3 g/L malt extract. For natamycin production, the strain was cultured in a fermentation medium (20.0 g/L soy peptone, 4.5 g/L yeast extract, 2.0 g/L NaCl, 1.0 g/L MgSO_4_, and 60.0 g/L glucose). The same media were supplemented with thiostrepton when used to culture *S. gilvosporeus* strain DE13. *Escherichia coli* ET12567 (pUZ8002) was used as a donor in intergeneric conjugations. *E. coli* BL21 (DE3) was used as a host for protein expression. The *E. coli* strains were cultured in lysogeny broth, containing 100 µg/mL kanamycin when necessary. The strains, plasmids, and primers used in this work are listed in Table S1.

### Transcriptomic analyses

For transcriptome sequencing and analysis, mycelia of *S. gilvosporeus* F607 cultured in different media were harvested after 24, 60, and 120 h. The transcriptomes were sequenced using a HiSeq 3000 sequencer (Illumina) at RibBio Corporation (Shenzhen, China). The transcript level of each gene was normalized by the number of reads per kilobase of transcriptome per million mapped reads (RPKM). Differentially expressed genes were selected using the Audics program with parameters |log_2_ fold-change| > 1 and *q*-value < 0.001.

### Construction of *sgnE* and *oxyR* deletion mutants and complementation strains

*sgnE* and *oxyR* deletion mutants strains were constructed by homologous recombination as previously (45). The whole *sgnE* gene was amplified with primers *sgnE* R-F and *sgnE* R-R using *S. gilvosporeus* F607 genomic DNA as the template (Table S2). The region downstream of the *sgnE* gene was amplified with primers *sgnE* L-F and *sgnE* L-R. Then, the two fragments were digested with *Xba*I and joined together using T4 DNA ligase. The fusion gene was cloned into plasmid pJTU1278 to construct pJTU1278-ΔsgnE using Gibson Assembly Master Mix (New England BioLabs, Ipswich, MA, USA). pJTU1278-ΔsgnE was transformed into *E. coli* ET12567 containing pUZ8002 and conjugated into *S. gilvosporeus* F607, as described by Wang et al. (24) Positive conjugants were selected using 10 µg/mL thiostrepton and 20 µg/mL streptomycin as resistance screening markers. Successful conjugants were confirmed by colony PCR with primers *sgnE* V-F1 and *sgnE* V-R1 and by DNA sequencing; the resulting strain was named *S. gilvosporeus* DE13. The *ermE* promoter region (*p_ermE_*) was amplified from pUC57, which is preserved in our laboratory (24). The *p_ermE_* and *sgnE* fragments were both digested with *Xba*I and joined together using Gibson Assembly Master Mix, and then, the joined fragment was cloned into pMS82; the resultant plasmid was transferred into *S. gilvosporeus* 607 to obtain *sgnE*-complemented *S. gilvosporeus* strain CE13, which was selected using 50 µg/mL hygromycin as a resistance screening marker. The *oxyR* deletion mutant *S. gilvosporeus* DO7 and its complemented mutant *S. gilvosporeus* CO7 were constructed using analogous procedures. Primers for *oxyR* mutation and complementation were listed in Table S2.

### Expression and purification of SgnE and OxyR

The *sgnE* gene was amplified from *S. gilvosporeus* F607 genomic DNA using the primer pair *sgnE* His-F/R (Table S2) and then cloned into pMD18T to generate pMD18T-sgnE. After confirmation by DNA sequencing, *sgnE* from pMD18T-sgnE was cloned into the expression vector pET-15b to construct pET-sgnE, which was transformed into *E. coli* BL21 (DE3) to obtain *E. coli* BL21/pET-sgnE. When *E. coli* BL21/pET-sgnE was cultured to an OD_600 nm_ value of 0.6–0.8, 0.1 mM isopropyl-d-1-thiogalactopyranoside was added to induce SgnE expression. His_6_–SgnE was purified by Ni-NTA chromatography (Sangon Biotech, Shanghai, China) (46). Construction of recombinant *E. coli* BL21/pET-OxyR and expression and purification of OxyR were performed using analogous procedures. Two fragments (amplified using the primers C212D-F and C212D-R) and pMD18T were linked using Gibson Assembly Master Mix and assembled with pET-15b to obtain the Cys212Asp variant of OxyR, following the same procedures.

### Enzyme assay and H_2_O_2_ quantification

The enzyme activities of SgnE from *E. coli* and *S. gilvosporeus* swjs-801 were determined by a colorimetric method with cholesterol, stigmasterol, and ergosterol as substrates as described by Wang et al. (24). Solution A (3 mL; 1 mmol/L 4-aminoantipyrine, 6 mmol/L phenol, 7,000 U/L peroxidase, 25 mmol/L potassium phosphate buffer, pH 7.5) was heated at 37°C for 3 min and then mixed with 0.02–0.10 mmol/L H_2_O_2_ for 5 min. The absorbance of the mixture was determined at 500 nm to establish a standard curve. Aliquots of shaken flask or bioreactor cultures were centrifuged at 10,000 × *g* for 20 min, and then, 1 mL of the supernatant was retained for enzyme assays. Solution A (3 mL) and 150 µL solution B (8.26 g/L cholesterol, stigmasterol, or ergosterol and 4.26% Triton X-100, dissolved in isopropanol) were heated at 37°C for 3 min and mixed with 50 µL fermentation supernatant, and the mixture was incubated for 5 min. The reaction mixture was boiled and then cooled on ice before determining the absorbance at 500 nm. Enzyme activity was calculated as enzyme activity (U/mL) = *C* × (*V*1/*V*2) × *N* ÷ *T*, where *C* is the H_2_O_2_ concentration determined from the standard curve, *V*1 is the total volume of the enzymatic reaction mixture, *V*2 is the volume of fermentation supernatant, *N* is the dilution ratio of fermentation supernatant, and *T* is the reaction time.

### Effect of H_2_O_2_ on natamycin production

The intracellular H_2_O_2_ concentrations in *S. gilvosporeus* strains DE13, CE13, and F607 were determined every 12 h for 120 h during fermentation using a Hydrogen Peroxide Assay Kit (Beyotime Biotech, Shanghai, China). Bioassays and high-performance liquid chromatography (HPLC) analysis of natamycin production were performed as follows. *S. gilvosporeus* DE13, CE13, and F607 were each cultured in seed medium at 29°C with shaking at 220 rpm for 16 h. Then, 1.5 mL of the culture was inoculated into a 250-mL flask containing 30 mL fermentation medium and cultured at 29°C with shaking at 220 rpm. The wet cell weight and natamycin production were determined every 12 h, as described previously (24, 32). H_2_O_2_ (100 µM) was added into the fermentation broth of *S. gilvosporeus* DE13 at 60 h, and natamycin production was determined 6 and 12 h later. Quantitative determination of natamycin was performed by HPLC analysis as described by Wang et al. (24)

### Real-time quantitative PCR analysis

Total RNA isolation and RT-qPCR were performed as described previously (47). Mycelia of *S. gilvosporeus* F607 and *S. gilvosporeus* DO7 were harvested from fermentation liquid at 24, 72, 120, 168, and 216 h and then rapidly frozen in liquid nitrogen. Total RNA was extracted from mycelia using an RNA extraction kit (SBSBIO, Beijing China) according to the manufacturer’s protocol and then treated with Turbo DNA-free reagents (ABI Ambion, Austin, TX, USA) to remove residual chromosomal DNA. cDNAs were synthesized using random hexamer primers (pdN6, Amersham Pharmacia Biotech, Buckinghamshire, England), M-MLV reverse transcriptase (Invitrogen, Carlsbad, CA, USA), and dNTPs (Roche, Basel, Switzerland). RT-qPCR assays were performed on a Roche Light Cycler 480 instrument using SYBR Green Mix (Toyobo, Osaka, Japan). Relative quantities of cDNA were normalized to the amounts of the 16S rRNA gene. For RT-qPCR assays, experiments were conducted in triplicate. The primers used are listed in Table S3.

### Analysis of OxyR redox status *in vivo* and *in vitro*

Aliquots of *S. gilvosporeus* CO7 cultures (OD_600 nm_ = 0.4) were taken with prewarmed pipettes and mixed with ^1^/_10_th vol of 100% trichloroacetic acid (TCA). Precipitated proteins were collected by centrifugation at 10,000 × *g* for 10 min. After the complete removal of the supernatant, the pellet was dissolved in AMS buffer to an OD_600 nm_ value of 10. Alkylation was performed at 37°C for a minimum of 2 h. Subsequently, aliquots (10 µL) of the alkylated samples were subjected to non-reducing SDS-PAGE. After electrophoresis, the separated proteins were blotted onto nitrocellulose membranes and probed with polyclonal antibodies against OxyR. The bound antibodies were detected using a western blotting kit.

For *in vitro* assays, samples of His_6_–OxyR were incubated in transcription buffer (pH 7.0) containing dithiothreitol at room temperature for a minimum of 24 h in an anaerobic chamber (Coy Laboratory Products, Ann Arbor, MI, USA). An aliquot (1.0 mL) was removed and acidified with ^1^/_10_th vol of 100% TCA. The remainder of the sample was treated with H_2_O_2_, and then, aliquots were precipitated with TCA at the indicated time points. The TCA-precipitated samples were removed from the anaerobic chamber and collected by centrifugation. The precipitated proteins were washed once with cold 10% TCA and collected by centrifugation. The pellet was redissolved and alkylated as described above. Samples were subjected to non-reducing SDS-PAGE.

### Molecular simulation analyses

Protein sequences were aligned using CLUSTALW2 at http://www.expasy.ch. Sequences were obtained using the protein search algorithm at the National Centre for Biotechnology Information (NCBI). Conserved domains of SgnR were analyzed by CD-Searches of the NCBI. A homology model of OxyR was; constructed using Robetta (https://robetta.bakerlab.org/). Multiple sequence comparison was carried out using Clustal Omega (48) and ESPript software (49). A homology model of OxyR (and the Cys212Asp variant) was constructed using Robetta and Discovery Studio 2.0 (50). The model stereochemical structure of OxyR and the DNA sequences of all intergenic regions in the natamycin biosynthesis gene cluster were used as the accepter and ligands, respectively, in molecular docking analyses. Molecular docking analyses of OxyR and DNA were performed using the ZDOCKER protocol of Discovery Studio 2.0 (50). Allosteric sites were predicted using AlloSitePro (2016) (51).

### EMSAs

A fragment of 150–300 bp was amplified from the intergenic region of *sgnM* and *sgnR* using *S. gilvosporeus* F607 genomic DNA as the template. The amplified DNA fragments were labeled at the 3′-end with biotin-11-UTP using a Biotin 3′ End DNA Labeling Kit (Thermo Scientific, Waltham, MA, USA). Non-specific cold probes (poly dI/dC) were added to control reaction mixtures as competitors. Electromobility shift assays (EMSAs) were carried out as described previously (52). To confirm the conserved DNA sites binding with OxyR, mutations of *p_sgnM_* were made (Table S2).

### Microscale thermophoresis

p*_sgnM_* was used as ligands, and different redox status OxyR [prepared by excess dithiothreitol and H_2_O2 (42, 52)] were used as targets. The RED-NHS protein labeling kit was used to label the empty proteins. The DNA fragment was continuously diluted for 16 gradients by reaction buffer [50 mM HEPES (pH 7.4), 0.05% Tween 20]. Mixture was incubated at room temperature for 20 min. Thermal swim signal of the mixture was measured by Monolith NT.115 Capillary. Affinity constant Kd was analyzed by using Nano Temper analysis software.

### Co-immunoprecipitation assays

For co-immunoprecipitation (Co-IP) assays, proteins were incubated with anti-OxyR antibody, anti-SgnR antibody, or control IgG overnight with the protein A/G agarose beads. The complexes were washed three times and resuspended in 2 × SDS loading buffer. The immunoprecipitated proteins were eluted from the beads by incubation at 95°C for 5 min. The eluted proteins were detected by immunoblotting after separation by SDS-PAGE.

## RESULTS

### *sgnE* and *oxyR* show maximum transcription level change during sterol-stimulated natamycin biosynthesis in *S. gilvosporeus*

**Fig 1:**
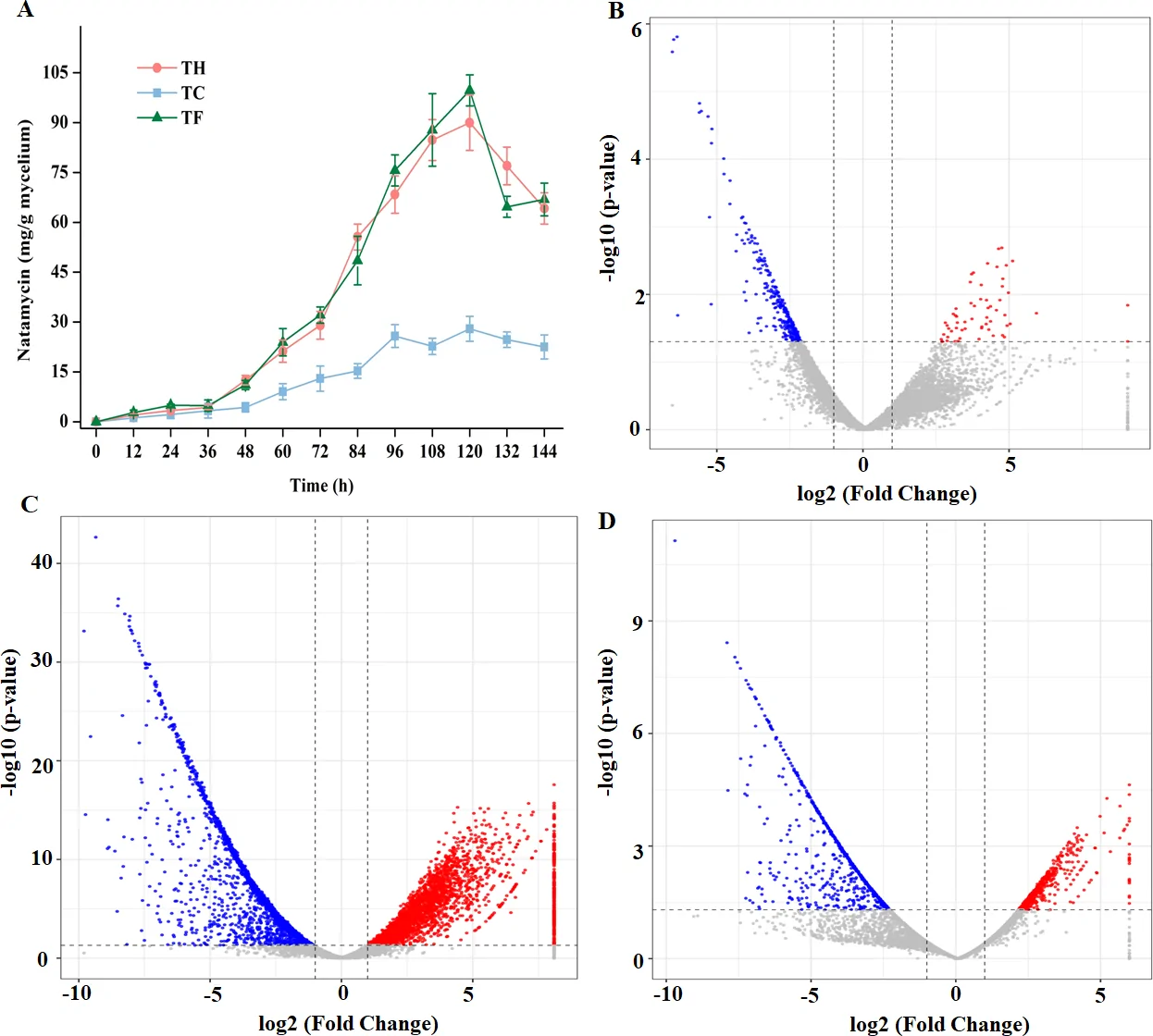
Influence of sterols on natamycin production. (**A**) Natamycin production by *S. gilvosporeus* with different sterols as substrates in the media (TH, control; TC, chopped sterols; TF, fertile sterols). (**B–D**) Volcano maps of differentially expressed genes in TC group compared with TH at 24, 60, and 120 h of culture, respectively. Red dots represent up-regulated genes, blue dots represent down-regulated genes, and gray dots represent non-differentially expressed genes.

Cholesterol, the main sterol in vertebrates, has been reported to be an effective elicitor of natamycin production (24). Here, we investigated whether the main sterols in plants and fungi, ergosterol and stigmasterol respectively, also trigger natamycin production in *S. gilvosporeus*. When *S. gilvosporeus* F607 was cultured in TC medium (defined in the Materials and Methods), which contained 2.0 g/L stigmasterol and 0.45 g/L ergosterol, the maximum production of natamycin was 27.92 mg/g mycelium. The production of natamycin reached its highest level, 91.3 mg/g mycelium, in TH medium, which contained 20 g/L ergosterol and 4.5 g/L stigmasterol. However, doubling the concentration of sterols to produce TF medium did not further promote natamycin production compared with the TH group (Fig. 1A).

To investigate how sterols stimulate natamycin production in *S. gilvosporeus* F607, we conducted transcriptomic and real-time quantitative PCR (RT-qPCR) analyses of samples collected at three points during natamycin synthesis after the initiation of culture (prophase at 24 h, rapid phase at 60 h, and late phase at 120 h). Transcriptome analyses (SRA data Accession NO: PRJNA949275) revealed that, in TC medium, which contained low concentrations of ergosterol and stigmasterol, 60 genes were upregulated and 283 genes were downregulated at 24 h compared with their respective transcript levels in TH medium (Fig. 1B). When natamycin began to be synthesized rapidly in the TC group at 60 h, the numbers of up- and downregulated genes increased to 1,941 and 2,816 compared with the TH group (Fig. 1C). When the natamycin synthesis rate slowed down at 120 h, there were 831 and 597 up- and downregulated genes, respectively, in the TC group compared with the TH group (Fig. 1D).

The differentially expressed genes were subjected to Gene Ontology (GO) term enrichment and Kyoto Encyclopedia of Genes and Genomes (KEGG) pathway analyses. The GO terms significantly enriched among differentially expressed genes were mainly in the categories “proton transmembrane transport” at 24 h (Fig S1A through C), “organonitrogen compound biosynthetic process” at 60 h, and “translation” at 120 h. The KEGG pathway analysis showed that many of the differentially expressed genes at 24 h were involved in “oxidative phosphorylation,” while those at 60 and 120 h were involved in “ribosome” (Fig S1D through F). The KEGG analysis showed that the term “type I polyketide structures,” which includes polyketide synthase (PKS) genes in the natamycin biosynthesis gene cluster, was enriched in differentially expressed genes at 24 and 60 h but not at 120 h. Moreover, genes in the “oxidation-reduction process” term and all the genes in the natamycin biosynthesis gene cluster showed significant differences in transcript levels between the TC and TH groups (Fig S1).

Thus, compared with the TC group, the TH group showed upregulation of genes associated with natamycin biosynthesis and the antioxidant system (Table S4). The transcript levels of PKS genes (*sgnS*0–*sgnS*4) were higher in the TH group than in the TC group (by 6.09- to 16.72-fold at 24 h, by 25.40- to 157.76-fold at 60 h, and by 1.54- to 4.67-fold at 120 h). Genes related to mycosamine formation and attachment to macrocyclic aglycones (*sgnJ*, *sgnC*, and *sgnK*) were also upregulated, especially in the rapid phase of natamycin biosynthesis (Table S4). Antioxidant system gene *oxyR*, in the TH group, upregulated to 2.54-fold at 24 h and 1.34-fold at 60 h but downregulated to 0.8-fold at 120 h (Table S4). This variation tendency is consistent with changes in natamycin synthesis rate in F607 [studied previously (45)]. To confirm the transcriptomic results, RT-qPCR was performed for representative genes. The RT-qPCR results were generally consistent with the transcriptomic results, except for RS10405 at 24 h (Fig. S2).

### SgnE catalyzes the production of H_2_O_2_ from sterols which provide a ROS signal

**Fig 2:**
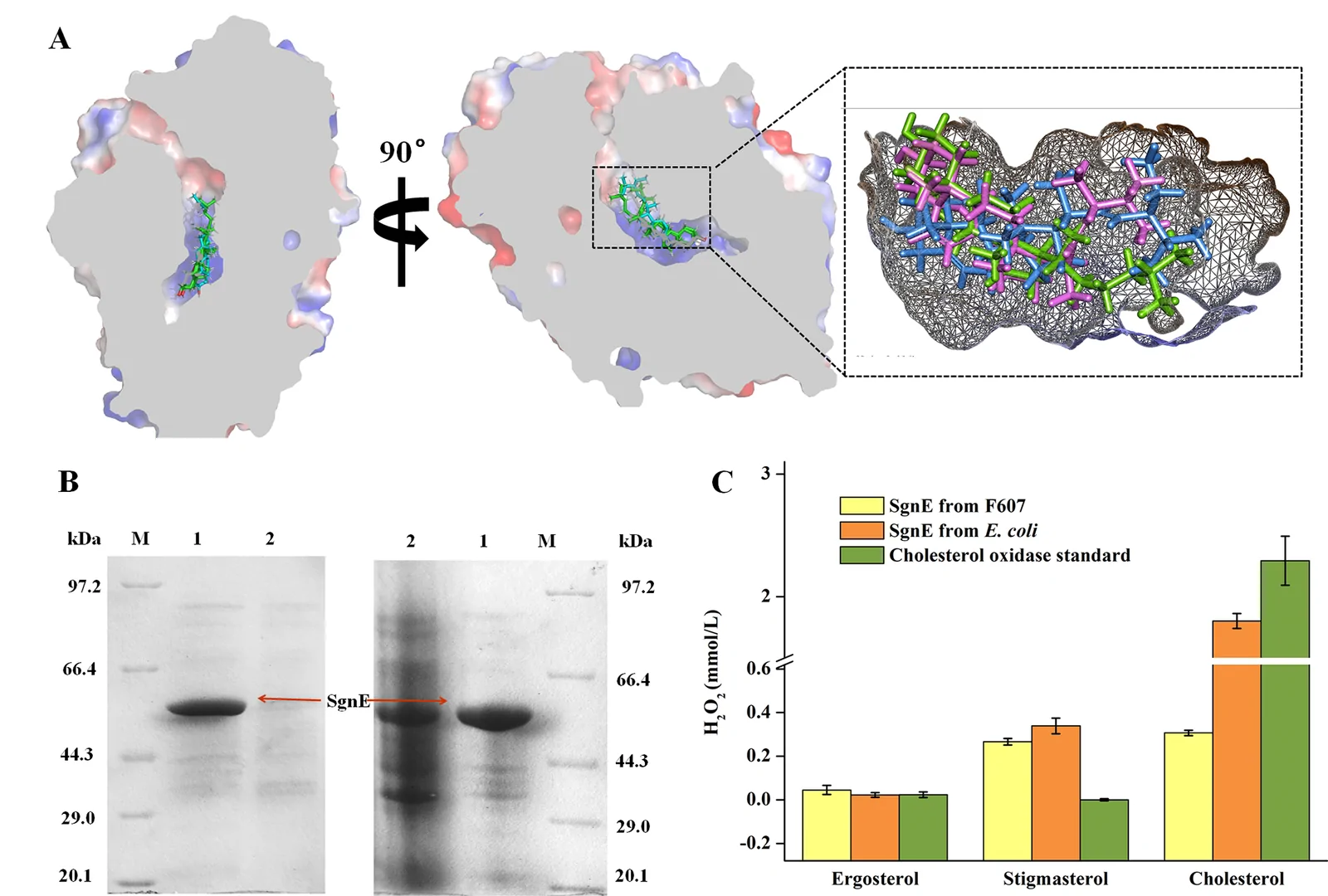
SgnE-mediated oxidation of sterols to generate H_2_O_2_. (**A**) Enlarged view of substrate-binding cavity. Gridlines indicate outline of cavity. Cholesterol, ergosterol, and stigmasterol are shown in blue, carmine, and green, respectively. (**B**) SgnE expression in different strains. Left, SgnE expressed in *S. gilvosporeus* swjs-801; Right, SgnE expressed in *E. coli* BL21 (DE3). 1, eluent liquid from Ni-NTA column; 2, permeate liquid from Ni-NTA column. (**C**) Detection and quantification of H_2_O_2_ by different cholesterol oxidase toward cholesterol, ergosterol, and stigmasterol.

Much debate has focused on the function of the cholesterol oxidase encoded by *sgnE* in the natamycin biosynthesis gene cluster in *Streptomyces*. To investigate its function in detail, we compared SgnE from *S. gilvosporeus* F607 with other cholesterol oxidases from *Streptomyces* (Fig. S3). A TAT (Tat/SPI)-type signal peptide for secretion (orange box), three repeating glycine residues (GXGXG) for interactions with FAD cofactor (53) (green box), and three residues (Ala403, Pro491, and Asn529) for catalysis (blue shading) were conserved in these cholesterol oxidases. To determine whether *Streptomyces* cholesterol oxidases degrade sterols as components of the “sterol cycle” in the natural environment, we selected three sterols, ergosterol, stigmasterol, and cholesterol (the main sterols in plants, fungi, and vertebrates, respectively) (6), for molecular simulation analyses. The tertiary structure of *S. gilvosporeus* F607 SgnE was modeled using structure PDB 4XWR (54) as the template (Fig. S4A). Ergosterol, stigmasterol, and cholesterol are structural analogs. The active site of the enzyme was buried in an amphipathic environment (Fig. S4B) that was large enough to accommodate the four-ring sterol structures. The enzymatic reaction cavity was connected to the loop cavity of the substrate-binding domain to form a central channel in SgnE, from the C-terminus to the N-terminus (Fig. 2A). In SgnE, the amino acid residues Tyr63, Val161, Val260, Ile261, Gly331, Tyr489, His490, Pro491, Pro529, and Ile533 formed the substrate-binding cavity. In this model, the 3β-hydroxyl group of the sterols was oxidized and interacted with the ketone of glycine (Gly331), and His490 acted as a general base catalyst during oxidation and an electrophilic catalyst during isomerization (Fig. S4C-E).

To verify the relationship between SgnE activity and H_2_O_2_ generation, SgnE from *S. gilvosporeus* F607 (as positive control) was cloned and expressed in *S. gilvosporeus* swjs-801 (24) and *Escherichia coli* BL21 (DE3) (Fig. 2B). The recombinant *S. gilvosporeus*-SgnE and *E. coli-*SgnE could successfully oxidize cholesterol, ergosterol, and stigmasterol to generate H_2_O_2_ (Fig. 2C). Thus, catalytic ability of SgnE in degrading sterols to form H_2_O_2_ , which is a signaling molecule in organisms (26), provides a possibility of regulating natamycin biosynthesis via its enzymatic product indirectly, rather than protein directly.

### Exogenous H_2_O_2_ alleviates the inhibition of natamycin production following *sgnE* deletion

**Fig 3:**
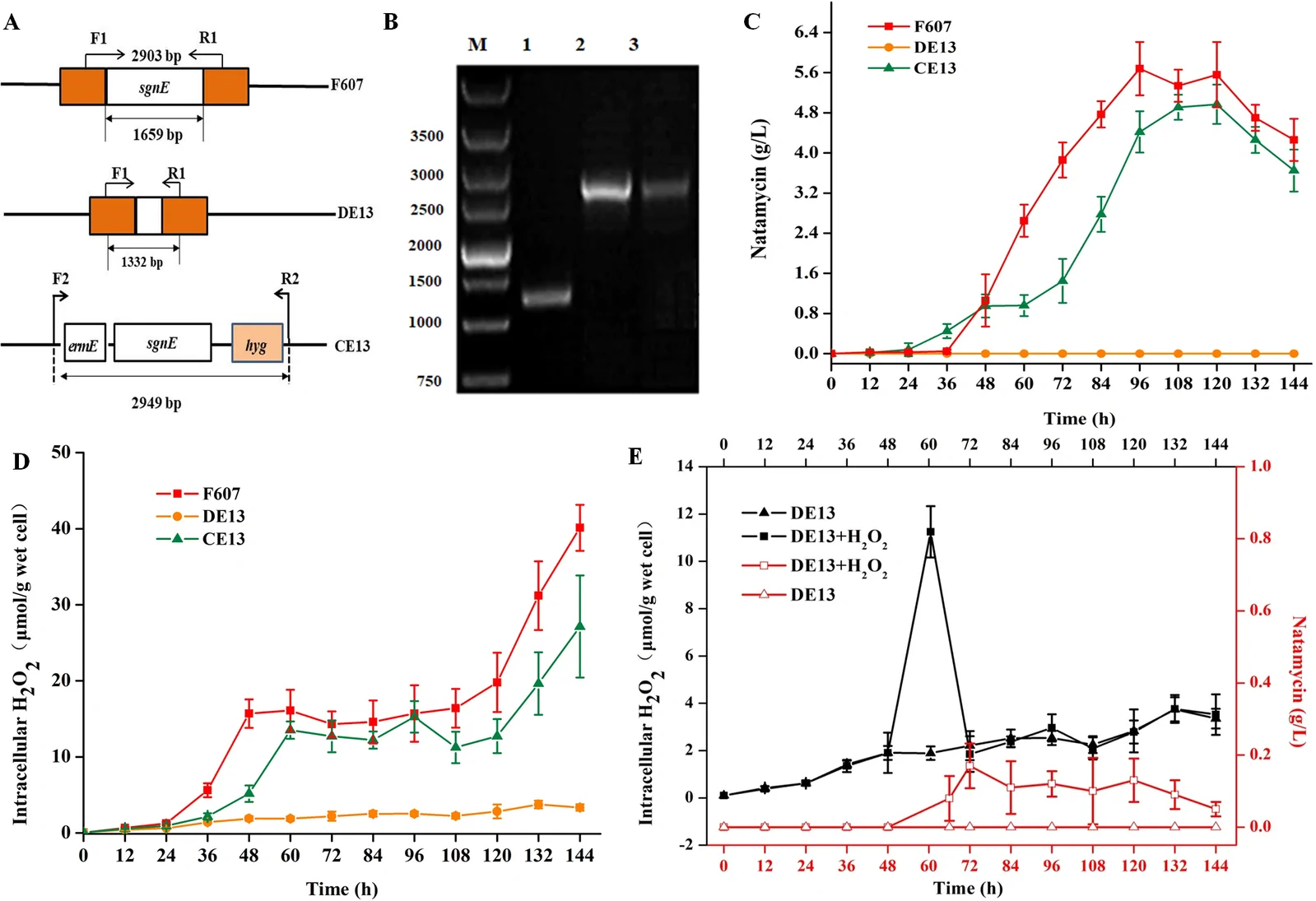
Exogenously addition of H_2_O_2_ restores natamycin production in the *sgnE*-deletion strain. (**A**) Schematic diagrams of construction of mutant strains with deleted *sgnE* gene (*S. gilvosporeus* DE13) and complemented *sgnE* gene (*S. gilvosporeus* CE13) in the *S. gilvosporeus* F607 background. F, forward primer; R, reverse primer. (**B**) PCR verification of DE13 and CE13 mutant strains. Lane 1, DE13; lane 2, CE13; lane 3, control F607. (**C**) Natamycin production by *S. gilvosporeus* F607 and its mutant strains DE13 and CE13 during fermentation in liquid medium. (**D**) Intracellular H_2_O_2_ concentrations in *S. gilvosporeus* F607 and its mutant strains DE13 and CE13 during fermentation in liquid medium. In (**C**) and (**D**), results are the mean ± standard deviation (SD) from triplicate experiments. (**E**) Restoration of natamycin production by *S. gilvosporeus* DE13 with *sgnE* deletion by addition of H_2_O_2_.

From *S. gilvosporeus* F607, we constructed the *sgnE*-deletion strain *S. gilvosporeus* DE13 and the *sgnE*-complemented strain *S. gilvosporeus* CE13 (Fig. 3A). Compared with the parental strain *S. gilvosporeus* F607, *S. gilvosporeus* strains DE13 and CE13 showed no obvious phenotypic changes when cultured in seed medium (NTZ), minimal medium (MM), or ISP2 (international *Streptomyces* project medium) (Fig. S5), indicating that SgnE is not essential for cell growth. However, *S. gilvosporeus* DE13 could not produce natamycin. Compared with *S. gilvosporeus* F607, the SgnE-complemented strain *S. gilvosporeus* CE13 had slightly low natamycin production (Fig. 3C).

During the natamycin synthesis stage of culture in fermentation medium (36–120 h), *S. gilvosporeus* F607 produced intracellular H_2_O_2_ at 5.62–19.80 µmol/g wet cell. In the *sgnE-*deleted strain *S. gilvosporeus* DE13, the H_2_O_2_ concentration was decreased to 1.41–2.83 µmol/g. *S. gilvosporeus* CE13, the *sgnE* complementation strain, produced H_2_O_2_ at a similar concentration to that produced by strain F607 (5.17–19.65 µmol/g) (Fig. 3D). When the intracellular H_2_O_2_ concentration in *S. gilvosporeus* F607 and CE13 presented a sharp increase at about 120 h of culture, natamycin production was decreased comparing with the earlier time points when the H_2_O_2_ concentration was lower. When the intracellular H_2_O_2_ concentration was 5.62 µmol/g at 36 h in *S. gilvosporeus* F607, or 5.17 µmol/g in *S. gilvosporeus* CE13 at 48 h, which were lower than 6.0 µmol/g, the biosynthesis of natamycin was inactivated (Fig. 3C and D).

To verify the relationship between natamycin synthesis and the intracellular H_2_O_2_ concentration, 100 µM H_2_O_2_ was added into the fermentation broth of *sgnE*-deleted strain *S. gilvosporeus* DE13 at 60 h. The intracellular H_2_O_2_ concentration increased to 11.25 µmol/g after 30 min, and natamycin could be detected with a yield of 0.08 mg/L after 6 h. Natamycin then continuously accumulated and its yield reached 0.17 g/L after 12 h. Similar phenomenon was not detected in the DE13 strain (Fig. 3E). These results indicate that the inhibitory effect of *sgnE* deletion on natamycin production could be partly alleviated by the addition of exogenous H_2_O_2_, suggesting that SgnE-mediated intracellular H_2_O_2_ production regulates natamycin biosynthesis in *S. gilvosporeus* F607.

### H_2_O_2_-sensing transcriptional regulator OxyR controls *sgnM* gene expression

**Fig 4:**
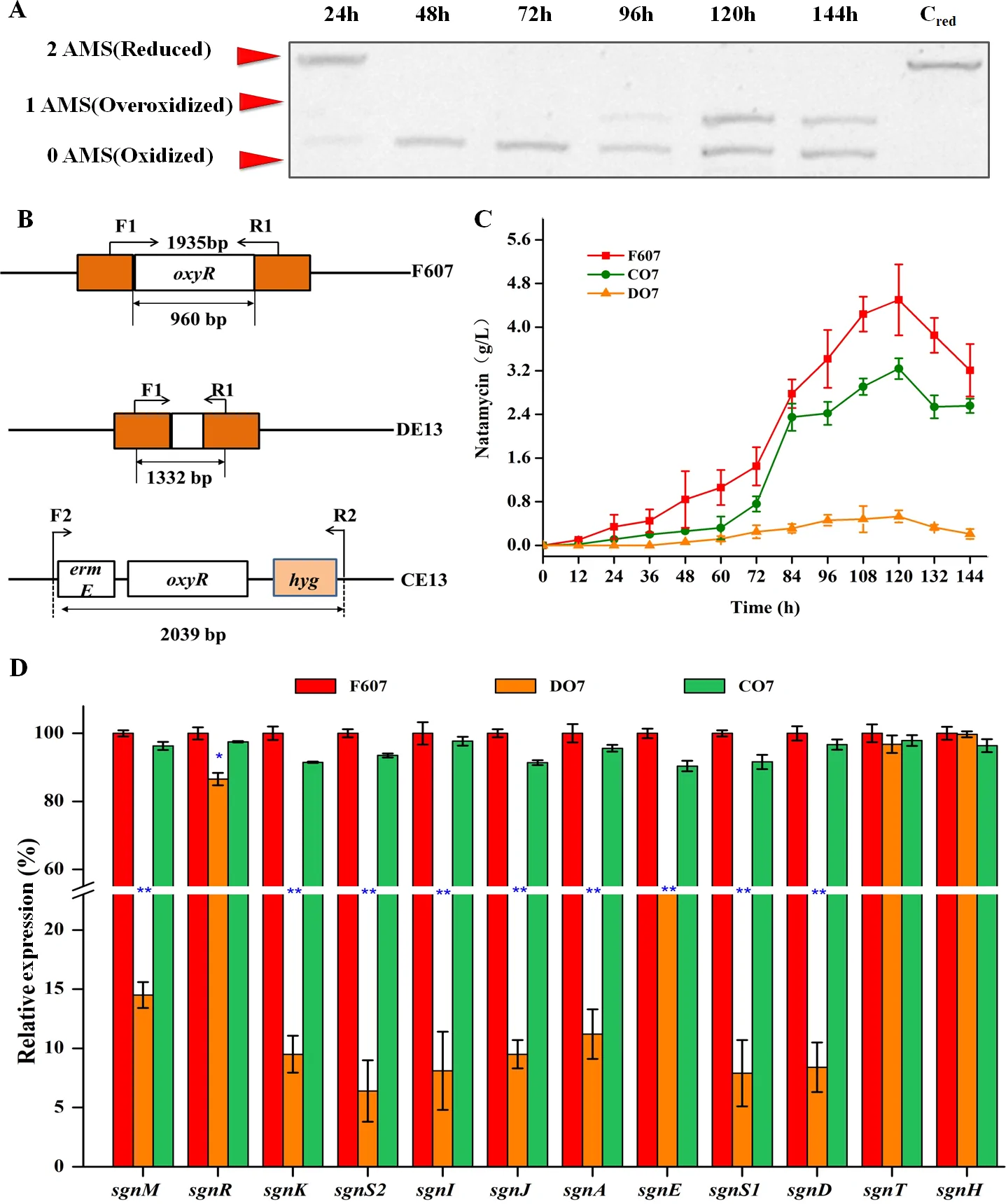
H_2_O_2_-sensing transcriptional regulator OxyR controls expression of genes in natamycin biosynthesis gene cluster. (**A**) Amounts of reduced, oxidized, and overoxidized OxyR at different stages of fermentation. (**B**) Schematic diagrams of *oxyR* mutant construction. F, forward primer; R, reverse primer. (**C**) Natamycin production at indicated times in cultures grown in the liquid fermentation medium. Results are the mean ± SD from triplicate experiments. (**D**) Transcript levels of genes from each transcriptional unit in *S. gilvosporeus* (parental strain), DO7 (*oxyR* deletion mutant), and CO7 (*oxyR* complementation mutant).

The intracellular H_2_O_2_ concentration was closely related to natamycin biosynthesis, and therefore, the role of the H_2_O_2_-sensing transcriptional regulator OxyR in natamycin production was investigated in *S. gilvosporeus*. OxyR from *S. gilvosporeus* F607 was precipitated with TCA, alkylated with fresh AMS buffer [0.1% (wt/vol) SDS, 1 M Tris, 15 mM 4-acetamido-49-maleimidylstilbene-2,29-disulfonic acid (AMS), pH 8.0] to alkylate free thiols, and then subjected to non-reducing SDS-PAGE and immunoblotting using an anti-OxyR antibody. The OxyR from *S. gilvosporeus* F607 was visible as three bands after reacting with AMS (Fig. 4A), indicative of the three redox states of the protein.

An *oxyR*-knockout strain, *S. gilvosporeu*s DO7, was constructed (Fig. 4B). *S. gilvosporeu*s DO7 showed decreased natamycin production, 0.53 g/L at 120 h, 11.8% of that produced by the parental strain *S. gilvosporeu*s F607 (Fig. 4C). Then, *S. gilvosporeu*s strain CO7 was constructed, in which strain DO7 was complemented with *oxyR* using the integrative vector pMS82. In strain CO7, natamycin biosynthesis was restored to 3.24 g/L at 120 h, slightly less than that produced by *S. gilvosporeu*s F607 (Fig. 4C). The transcript levels of each transcriptional unit were detected by RT-qPCR in *S. gilvosporeu*s strain DO7 to investigate differences in the expression of the natamycin biosynthesis gene cluster upon *oxyR* deletion.

The transcript levels of the natamycin efflux pump gene *sgnH* and PI factor secretion gene *sgnT* showed no significant difference between *S. gilvosporeu*s strains DO7 and F607. However, the transcript level of the pathway-specific regulatory factor gene *sgnM* was decreased in *S. gilvosporeu*s DO7 to 16.3% of the level in the parental strain *S. gilvosporeu*s F607, while the transcript level of the other regulatory factor gene *sgnR* was only slightly decreased to 86.5% of that in *S. gilvosporeu*s F607. Other genes also showed decreased transcription upon disruption of *oxyR*. For example, the transcript levels of the type I modular PKS-encoding genes (*sgnS0*, *sgnS1*, and *sgnS2*) were dramatically decreased to only 6.4%–11.2% of their respective levels in *S. gilvosporeu*s F607. The transcript levels of other genes, including *sgnK*, *sgnI*, *sgnJ*, *sgnA*, *sgnE*, and *sgnD* in gene clusters controlled by SgnM, were also obviously decreased in strain DO7 to 9.5%–27.6% of their respective levels in strain F607 (Fig. 4D). These results indicate that OxyR activity is mediated by H_2_O_2_, and it regulates the expression of *sgnM*.

### OxyR consensus sequence interacts with the four motifs in the shared promoter region of P_*sgnMR*_

To evaluate the relationship between OxyR, natamycin biosynthesis, and the intracellular H_2_O_2_ concentration, the OxyR from *S. gilvosporeus* F607 (OxyR_Sgi_, WP_083107198.1) was compared with OxyRs from *E. coli* (OxyR_Eco_, AAA24257.1), *Mycobacterium leprae* (OxyR_Mle_, WP_010908684.1), *Xanthomonas campestris* (OxyR_Xca_, AAC45427.1), *Vibrio vulnificus* (OxyR_Vvu1_, ADV85339.1; and OxyR_Vvu2_, ADV87476.1), and other natamycin-producing *Streptomyces* strains (14). All the OxyRs from *Streptomyces* strains contained two cysteine residues, compared with seven in OxyR_Vvu2_, six in OxyR_Eco_ and OxyR_Vvu1_, four in OxyR_Mle_, and three in OxyR_Xca_. In all the OxyRs listed, two cysteines (residues Cys212 and Cys221 in OxyR from *S. gilvosporeus* F607) were conserved in an “LLL(D/E)(E/D)GH**C**LRDQxxxx**C**(H/F)” peptide fragment (Fig. S6). A homology model of reduced OxyR from *S. gilvosporeus* F607 was generated using Discovery Studio, based on 1I69 (Fig. S7A). Each monomer was composed of 11 α-helixes and 9 β-sheets (Fig. S7B). The dimer of DBD-interfaced homodimers forms a “wrench-like” conformation *via* interactions between the linker helixes of the two DBDs and creates an arch route for the DNA to traverse when binding (Fig. S7C).

**Fig 5:**
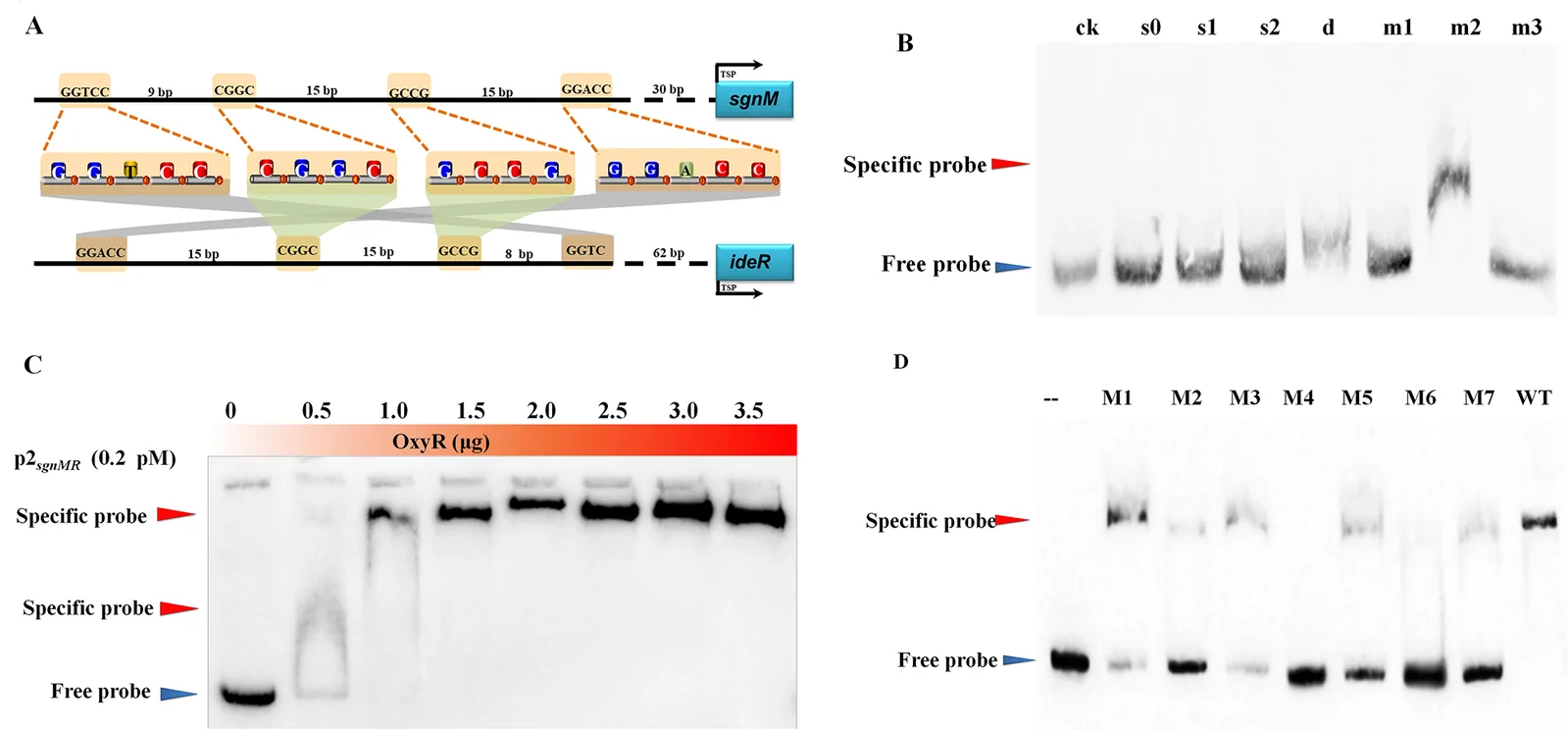
OxyR binds the promoter region of *sgnM*. (**A**) EMSAs of OxyR–p*_sgnM_* bidirectional promoter region using His6–OxyR protein. ck, control; s0, p*_sgnS0_*; s1, p*_sgnS1_*; s2, p*_sgnS2_*; d, p*_sgnD_*; m1, p1*_sgnM_*; m2, p2*_sgnM_*; m3, p3*_sgnM_*. (B) Competition tests to evaluate specific interaction between OxyR and p*_sgnM_*_-2_. (C) Binding sequences of OxyR within promoter region of *sgnM*. (D) Mutational analyses of predicted OxyR-binding sites in *sgnM* promoter. OxyR binding was tested in EMSAs using probes with wild-type or mutated sequences.

The predicted DNA-binding sequence of OxyR in the p*_sgnMR_* region, which is similar to the binding site for *ideR* in *S. avermitilis*, covered −30 to −85 bp upstream of the transcription start site of *sgnM* (Fig. 5A). To confirm the binding of OxyR to the promoter region of the natamycin pathway-specific transcriptional regulator gene *sgnM*, the binding of OxyR with p*_sgnM_* was analyzed using EMSAs. We tested biotin-labeled probes of the *sgnMR* promoter region, p*_sgnM_*_-1_–p*_sgnM_*_-3_, and, as negative controls, the promoter regions of *sgnS*0, *sgnS*1, *sgnS*2, and *sgn*D (p*_sgnS0_*, p*_sgnS1_*, p*_sgnS2_*, and p*_sgnD_*, respectively). Probe p*_sgnM_*_-2_, containing the predicted binding sequence of OxyR to p*_sgnM_*, was shifted in EMSA by the addition of purified oxidized OxyR (Fig. 5B). To evaluate the specific interaction between OxyR and the promoter region of *sgnM*, competition tests were performed using oligonucleotide probe p*_sgnM-_*_2_. A small amount of probe *sgnM*2 was shifted by 0.5 µg OxyR; most of the probe was shifted by 1.0 µg OxyR, and 1.5 µg OxyR was enough to shift all the *sgnM*2 probe (Fig. 5C).

Sequence analyses revealed that a sequence in p*_sgnM_*_-2_ was similar to a previously reported OxyR-binding site in the promoter of *ideR* in *S. avermitilis*. They shared the conserved sequence “GGT/ACC–15 nt–CGGC–15 nt–GCCG–9/8 nt–GGA/TCC.” The four parts of this palindromic sequence were named motifs 1–4. To validate the OxyR consensus-binding sequence, oligonucleotide probes of the predicted recognition sequences containing motif mutations were tested in EMSAs. Mutations of motif 1 (M1), motif 2 (M2), or motif 3 (M3), respectively, dramatically decreased the binding affinity of OxyR for p*_sgnM_* compared with the native sequence (Fig. 5D, lanes M1–M3). Importantly, mutation of motif 4 (M4) abrogated OxyR binding with p*_sgnM_* (Fig. 5D, lane M4). When the tests were conducted with mutations in both motifs 1 and 2 (M5) (Fig. 5D, lane M5), or in all of motifs 1, 2, and 3 (M7) (Fig. 5D, lane M7), binding was weaker than that detected with the wild-type sequence. The simultaneous mutation of all four motifs (M6) abrogated OxyR binding with p*_sgnM_* (Fig. 5D, lane M6). These findings suggest the importance of these motifs in the interaction between OxyR and p*_sgnM_*.

### Oxidized OxyR shows higher motif-binding affinity with p*_sgnM_* than reduced or overoxidized OxyR

Allosteric site predictions showed that the asymmetrical tetrameric OxyR contained four allosteric sites (Table S5). All the allosteric sites were in close proximity to the DBDs of OxyR. Eight cysteines (two in each monomer) are wrapped in the center of the tetramer and form an “H_2_O_2_ reaction pocket.” The frame of this H_2_O_2_ reaction pocket was formed by eight α-helixes (four α6 and four α8) (Fig. S8).

**Fig 6:**
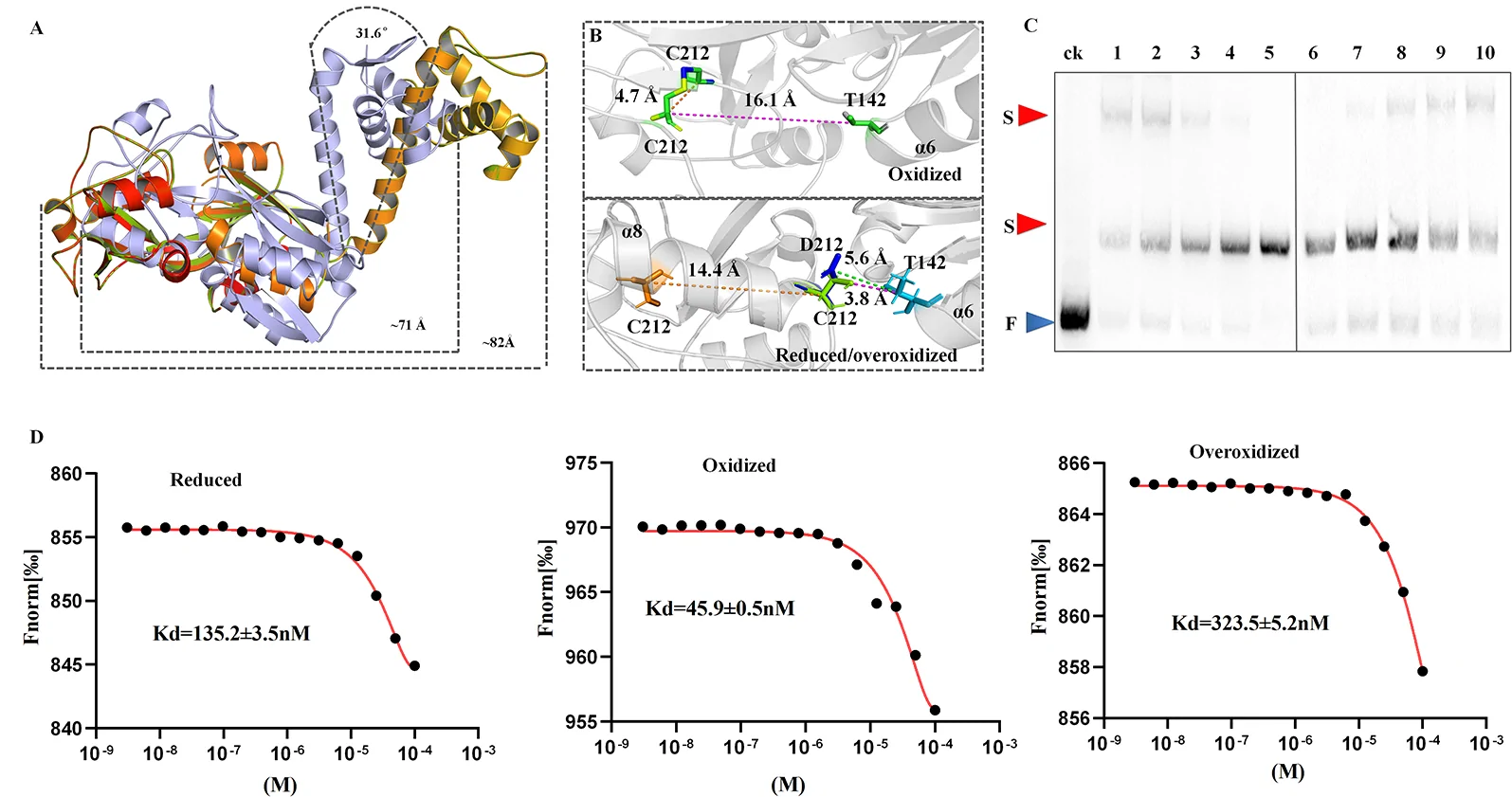
Redox states of OxyR induce conformational changes to affect its binding affinity with promoter region of *sgnMR*. (**A**) Modeled structures of OxyR in different redox states. Reduced, oxidized, and overoxidized OxyR are colored in red, orange, and gray. (**B**) Distances of α6 and α8 during conformations changes. (**C**) Binding of reduced, oxidized, and overoxidized OxyR with promoter region of *sgnMR*. ck, control. Lanes 1–4, oxidized OxyR treated with 1,000; 100; 50; and 10 µM dithiothreitol; lane 5, 100% oxidized OxyR; lanes 6–10, oxidized OxyR treated with 10; 50; 100; 500; and 1,000 µM H_2_O_2_. (**D**) Microscale thermophoresis results of different oxidation states with p*_sgnM_* DNA.

On oxidation, the diameter of the whole protein is shortened, and α4 rotated by 31.6° relative to its position in the reduced protein (Fig. 6A). In reduced OxyR, Cys212 and Cys221 are located at the ends of an α-helix in the RD, 14.4 Å apart (C_α_–C_α_). After OxyR was oxidized by H_2_O_2_, disulfide bond formation unfolded the helix, resulting in the formation of a short β-strand, and the C_α_–C_α_ distance between Cys212 and Cys221 shortened to 4.7 Å (Fig. 6B). Conformational changes in OxyR from reduction to oxidation are presented in Video S1. Overoxidized state of OxyR resulted in a conformation of helix α3 that was almost identical to that in reduced OxyR; the Cys212Asp mutation recovered the distance between Cys212 and Cys221 to 14.4 Å (Fig. 6B). The conformational change between the oxidized and overoxidized states of OxyR is presented in Video S2.

To investigate the effect of conformational changes of reduced, oxidized, and peroxidated OxyR on *sgnM* regulation, EMSAs were performed to investigate the binding of OxyR in different oxidation states with p*_sgnM_* DNA. Reduced and oxidized OxyR were prepared by dithiothreitol and H_2_O_2_ treatment, respectively. The overoxidized state of OxyR was mimicked by using a Cys212Asp mutant of OxyR. Reduced, oxidized, and overoxidized OxyR could all bind to the DNA sequence of p*_sgnM_*. However, the oxidized OxyR showed a higher affinity for p*_sgnM_* than the reduced and overoxidized states (Fig. 6C). This binding was confirmed by microscale thermophoresis. Kd of reduced, oxidized, and overoxidized OxyR binding with p*_sgnM_* were 135.2 ± 3.5 nM, 45.9 ± 0.5 nM, and 323.5 ± 5.2 nM, respectively (Fig. 6D). Affinity of different oxidation states binding with p*_sgnM_* is oxidized OxyR > reduced OxyR > overoxidized OxyR. Therefore, OxyR presented low, high, and low DNA-binding affinities with the *sgnM* promoter region in the reduced, oxidized, and peroxidated states, respectively. This indicates that the conformation of OxyR regulates natamycin biosynthesis and is dynamically variable in *S. gilvosporeus*.

### SgnR–OxyR complex cooperatively regulates *sgnM* transcription to control natamycin synthesis in response to H_2_O_2_ concentration

**Fig 7:**
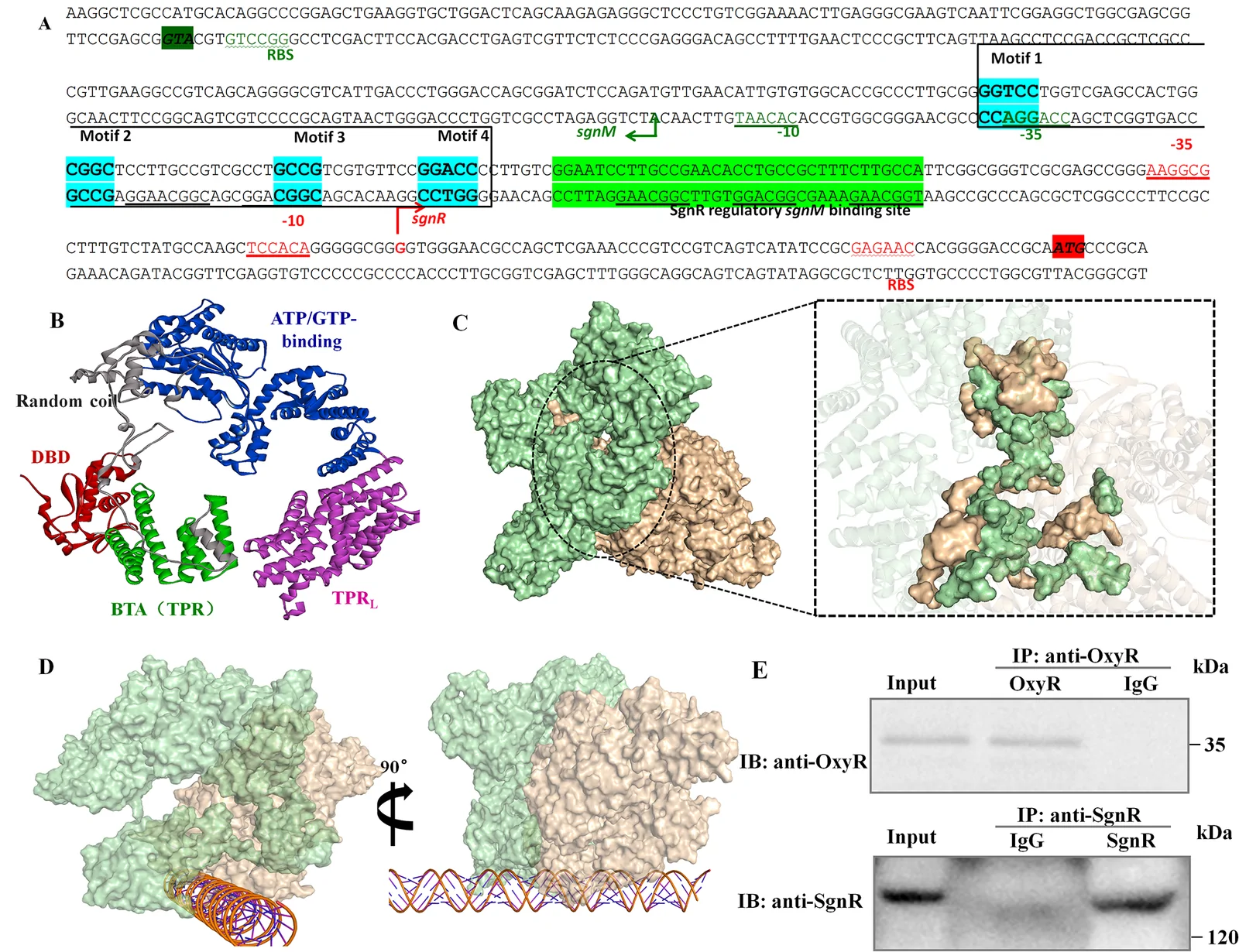
OxyR regulates *sgnM* in cooperation with SgnR. (**A**) Sequence arrangement upstream of *sgnM*. Putative −10 and −35 hexanucleotides are underlined. Transcription start point is indicated by a bent arrow and bold-type letter. Box labeled “RBS” indicates ribosome-binding sites. Start codon is shown in a red box. Shaded box indicates SgnR-binding site on *sgnM*. OxyR-binding site inverted repeat motifs are shown in bold and labeled Motif 1, 2, 3, and 4. (**B**) Tetrameric structure of SgnR from *S. gilvosporeus*. DBD, BTA, random coil, ATP/GTP-binding domain TPR-like domain are shown in red, green, gray, blue, and carmine, respectively. (**C**) Protein–protein complex comprising OxyR (brown) and SgnR (blue–green) and interaction surface. (**D**) Protein–protein–DNA complex comprising OxyR (brown), SgnR (blue–green), and p*_sgnM_*. (**E**) proteins were co-immunoprecipitated by OxyR and SgnR antibodies with IgG as negative control. The result showed that PDCD4 interacts with DTL.

SgnR binds to the *sgnM* promoter to activate its transcription, and then, SgnM activates transcription of eight genes in the natamycin biosynthesis cluster (14). The DNA-binding sequence of OxyR in the *sgnM* promoter region was “GGTCC–15 nt–CGGC–15 nt–GCCG–9 nt–GGACC.” The four parts of this palindromic sequence were named motifs 1–4, respectively. Motif 1 of this sequence covered the −35 bp region upstream of *sgnM* (Fig. 7A). Reduced and overoxidized OxyR adopt an extended conformation and turn off P*_sgnM_* by binding to elongated sequences masking the −35 region, while the contracted architecture of oxidized OxyR unmasks the −35 region of target gene *sgnM*. This is known as the “OxyR regulatory mode” (42). Notably, on *sgnM* promoter region, the OxyR-binding sequence was located 7 bp downstream of the SgnR-binding site, *via* which *sgnM* transcription was strictly controlled (55) (Fig. 7A).

Considering that the DNA-binding site of SgnR to *sgnM* was not contained in the −35 region of *sgnM*, which is not consistent with the regulatory mode of other (Streptomyces antibiotic regulatory protein) SARP proteins (56), we conducted molecular simulation analyses to explore the interaction between SgnR and OxyR. First, the conserved domains and architecture of SgnR were predicted. SgnR is an SARP regulator consisting of 1,184 amino acid residues in *S. gilvosporeus* F607. It was found to contain five domains, namely, an N-terminal DNA-binding domain, a bacterial transcriptional activation domain consisting of three-tetratricopeptide repeats (TPRs), a random coil, an ATP/GTP-binding domain, and a TPR-like domain (Fig. 7B). Molecular docking analyses showed that SgnR could combine with the OxyR tetramer. Interaction residues, located in DBD domain of DBD and the RD of OxyR and random coil of SgnR, respectively, combined four regions when the two regulatory proteins interact (Fig. S9). The random coil of SgnR was inserted into the cavity located between the DBD and the RD of OxyR, this combination surface presents a long and narrow shape (Fig. 7C). This combination made the DBDs of OxyR and SgnR arrange into a linear structure, resulting in the formation of an extended groove (Fig. 7C). The extended groove provided a gap to allow DNA containing the binding sites for SgnR and OxyR to pass through (Fig. 7D). Co-IP demonstrated that OxyR could interact with SgnR (Fig. 7E). Functional complementation of the SgnR–OxyR complex synergistically regulate the transcription of *sgnM* by masking or unmasking the −35 region of the *sgnM* promoter to control natamycin synthesis in response to ROS concentration although sequential order of OxyR–SgnR complexes needs a further investigation.

## DISCUSSION

In natural ecosystems, animals, plants, and microbes have evolved symbiotic interactions (57). The genus *Streptomyces* is an abundant source of secondary metabolites, including antibiotics, anticancer compounds, immunosuppressants, pigments, herbicides, and enzymes. Ecologically, as saprophytic soil bacteria, *Streptomyces* play key roles in the natural recycling of the cell walls of plants, fungi, and insects (4, 5). Sterols are a type of lipid that exist in membranes of diverse organisms from unicellular fungi and protists to multicellular animals and plants (6). In many multicellular organisms, sterols have another very important role in the production of signaling molecules such as steroids in vertebrates, brassinosteroids in plants, and ecdysteroids and molting hormones in invertebrates (6). Ecological and environmental conditions are believed to influence the metabolic capacity of bacteria to produce secondary metabolites (58). However, the role of *Streptomyces* in the “sterol cycle” and the influence of sterols on antibiotic metabolism in *Streptomyces* have rarely been investigated. Here, we show the function of sterols as elicitors that activate natamycin biosynthesis in *S. gilvosporeus*. Our results also show that the signaling molecule H_2_O_2_, generated from the degradation of sterols by cholesterol oxidase from *S. gilvosporeus*, regulates natamycin biosynthesis *via* the peroxide-sensing transcriptional regulator OxyR and pathway-specific regulator SgnR.

Natamycin is a 26-membered tetraene macrolide antifungal antibiotic that can be produced by *S. gilvosporeus*, *S. chattanoogensis* (10), *S. natalensis* (14), and *S. lydicus* (18). These strains contain natamycin biosynthesis gene clusters with different arrangements and members, but all of them contain a gene-encoding cholesterol oxidase, an enzyme that oxidizes cholesterol to produce H_2_O_2_ (19). Natamycin can be oxidized by H_2_O_2_ (35, 36). This contradiction indicates that a precise regulatory network balances natamycin biosynthesis and ROS homeostasis in natamycin-producing strains. Crosstalk between ROS homeostasis and natamycin metabolism was first proposed for *S. natalensis* ATCC 27448 (34). Consistent with previous evidence (19, 23), our results show that the cholesterol oxidase-encoding gene *sgnE* in the natamycin biosynthesis gene cluster is indispensable for natamycin biosynthesis in *S. gilvosporeus*. SgnE can oxidize at least three sterols, ergosterol, stigmasterol, and cholesterol (the main sterols in plants, fungi, and vertebrates, respectively) (6), to generate H_2_O_2_. In previous studies, the enzymatic activity of cholesterol oxidase was shown to be a key factor for natamycin production by deletion (19) or overexpression (24) analyses. Another study showed that no natamycin was produced when H_2_O_2_ or cholestenone was addedto the culture broth of a cholesterol oxidase gene mutant (23). However, according to our results, the concentration of H_2_O_2_ added in those experiments (10 pM to 10 nM) was insufficient to activate natamycin production. Additionally, in some previous reports on natamycin production, cholesterol oxidase was unable to interact with sterols because the latter were absent from the PMM medium that was used (which lacks yeast extract or malt extract). However, PMM contains diverse metal ions such as copper, manganese, and molybdenum, which can induce intracellular oxidative stress (59 – 61).

Cholesterol oxidase-encoding genes are also found in gene clusters for the biosynthesis of small polyene macrolide antibiotics including tetramycin (20), filipin (21), and rimocidin/CE-108 (22). The biosynthesis of polyene macrolide antibiotics *via* gene clusters that contain a cholesterol oxidase-encoding gene may be a pathway to regulate H_2_O_2_ homeostasis in the various strains that produce these compounds. Although H_2_O_2_ is better known for its cytotoxic effects, it is also an important regulator of eukaryotic (25) and prokaryotic (62) signal transduction. Our data indicate that *S. gilvosporeus* functions as a sterol-degrading bacterium in the “sterol cycle” to generate H_2_O_2_, which then elicits natamycin biosynthesis. This may be a common characteristic among these strains although more research is required to confirm this.

When ROS levels exceed safe limits, bacteria mount an inducible response, resulting in increased expression of ROS-detoxification enzymes and protective systems that repair oxidative damage (62). Global regulators, such as sigma factors, two-component regulatory systems, SoxR, OxyR, OhrR, and CatR–CatA, appear to be the main factors that respond to ROS in *Streptomyces* (26, 62). OxyR, a LysR family H_2_O_2_-sensing transcriptional regulator, is generally found in Gram-negative bacteria and also in a few Gram-positive bacteria (62). It is activated by disulfide bond formation between two cysteine residues. OxyR fulfills the definition of a peroxide receptor; in *E. coli*, it is activated by H_2_O_2_ at concentrations that just exceed cellular physiological levels (20 nM) but is below the threshold toxic level (estimated at >1 µM) (26, 41). In *V. vulnificus*, OxyR functions as a three-state redox switch that tightly regulates the expression of *prx2*, preventing the futile production of Prx2 in cells exposed to levels of H_2_O_2_ that are sufficient to inactivate it. Bang et al. reported that there are reduced, oxidized, and overoxidized states of OxyR. Cys206 in OxyR_Vvu1_ and OxyR_Vvu2_ (equivalent to Cys212 in *S. gilvosporeus* OxyR) was identified as the sensing residue that is overoxidized to S-sulfonated cysteine (Cys–SO_3_H) by high H_2_O_2_ concentrations *in vitro* and *in vivo* (42). The two cysteines of OxyR have the H_2_O_2_-sensing function in *Streptomyces*, and our experiments verified the different oxidation states of OxyR in *S. gilvosporeus*. We show that SgnE affects the production of natamycin *via* the OxyR oxidative stress-response system. Thus, we conclude that natamycin biosynthesis, a way to accumulate a reduced product, is one of the pathways to regulate H_2_O_2_ homeostasis.

In *E. coli*, there are >20 target genes of OxyR, including genes involved in H_2_O_2_ detoxification, heme biosynthesis, reductant supply, thiol-disulfide isomerization, Fe–S center repair, iron binding, and repression of iron and manganese import, as well as the small regulatory RNA OxyS (62). In *Streptomyces*, the target genes of OxyR include those encoding components of the alkyl hydroperoxide reductase system in *S. coelicolor* A3(2) (63), antioxidant enzymes, oxidative stress regulators (64), and the iron-response regulator IdeR in *S. avermitilis* (65). In the present work, we show that the DBD of OxyR can grasp the DNA of p*_sgnM_*. Together, our results show that the natamycin pathway-specific transcriptional regulator gene *sgnM* is a target of OxyR in *S. gilvosporeu*s and that the redox state of OxyR affects its binding to the promoter region of *sgnM*. The binding sequence of reduced and overoxidized OxyR, GGTCC–15 nt–CGGC–15 nt–GCCG–9 nt–GGACC, is similar to that of the *ideR* promoter region in *S. avermitilis* (65) and the “ATAGntnnnanCTAT–7 nt–ATAGntnnnanCTAT” sequences in the promoters of *oxyRS*, *katG*, and *ahpC* in *E. coli* and *Salmonella typhimurium*. According to previous results, the distance between DBD1 and DBD3 of OxyR in the reduced state is 120 Å, and this distance is shortened to 75 Å in the oxidized state; that is, the DBD1–DBD2 dimer and the DBD3–DBD4 dimer get closer by 45 Å following the transition from the reduced to the oxidized state (66). Thus, when OxyR is oxidized, it releases the “GGACC” motif and exposes the whole −35 region of the *sgnM* promoter in *S. gilvosporeus*. While, precise mechanism about OxyR regulation on *sgnM* and other ROS homeostasis targets should be verified by DNase I footprinting assay or protein–DNA complex crystallographic structural analysis in further studies.

SgnM, SgnR, and their homologs are pathway-specific transcriptional regulators. They share a bidirectional promoter region and are encoded within their respective biosynthesis gene clusters. Oxidation of (a) cysteine residue(s) in a protein prevents its alkylation by AMS (42, 43). Alkylation of one cysteine residue by AMS increases the molecular mass of the protein by 0.5 kDa (and alkylation of two increases it by 1 kDa), which can be detected by SDS-PAGE. In reduced *S. gilvosporeus* OxyR, two cysteine residues can be alkylated by AMS. If only one cysteine residue is alkylated by this reagent, it indicates that one of the two cysteine residues in OxyR is already oxidized, indicative of the overoxidized state of OxyR. The regulatory effect of OxyR on natamycin biosynthesis does not depend on its expression level, but on its oxidation state, because that affects its binding affinity to the target DNA. The oxidation state of OxyR is determined by the intracellular H_2_O_2_ concentration. Thus, the DNA (promoter) binding affinity and binding coverage of different redox states of OxyR control the expression of *sgnM* and contribute to the dynamic regulation of natamycin biosynthesis in response to ROS in *S. gilvosporeus*.

The SgnR homolog PimR binds to a main operator that contains three heptameric direct repeats of the consensus CGGCAAG sequence with 4 bp spacers, which seems to be the DBD-binding pattern that is conserved among SARP regulators (56). PimR also binds to a secondary operator with two heptameric repeats of the consensus sequence separated by a 3 bp spacer. The optimum number of repeats of the heptamer recognized by PimR is three, and at least one triplet is required at the operator to activate *pimM* transcription (56). Unlike SARP regulators that bind DNA overlapping the −35 hexamer of target promoters, SgnR binds to a sequence located upstream of that hexamer (55). Surprisingly, we found that the OxyR DNA-binding sequence includes the two heptameric repeats that were predicted to be the SgnR-binding site. Protein–protein interaction analyses indicated that SgnR cooperates with OxyR to regulate the expression of *sgnM*. The formation of an SgnR–OxyR protein–protein complex can mask or unmask the −35 region of the *sgnM* promoter depending on the oxidation state of OxyR, ultimately regulating the expression of *sgnM*.

**Fig 8:**
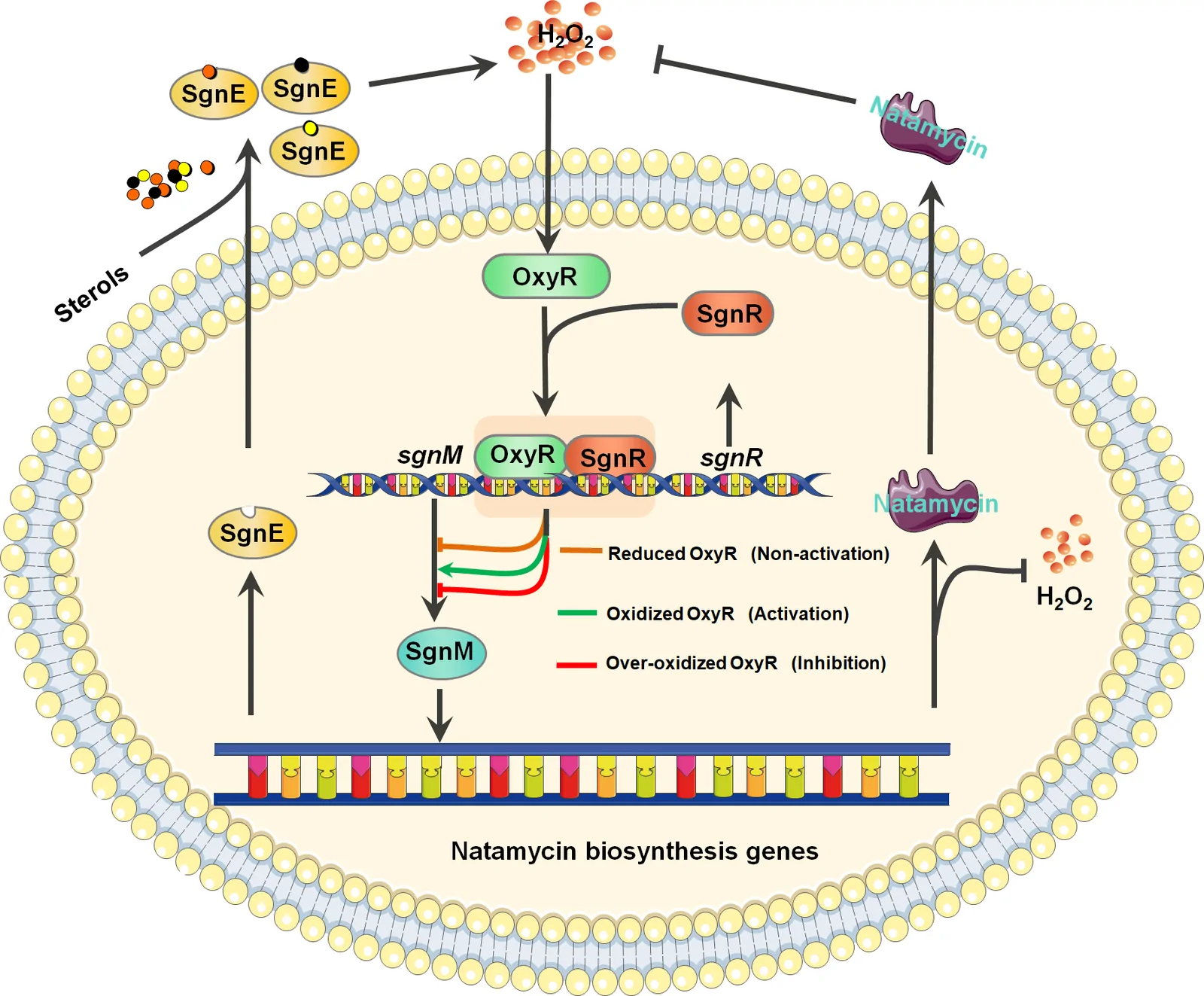
Dynamical regulation mechanism of efficient natamycin biosynthesis in response to oxidative stress by *Streptomyces*.

In summary, *Streptomyces* participates in the “sterol cycle” *via* cholesterol oxidase SgnE, whose coding gene *sgnE* is located in the natamycin biosynthesis gene cluster. SgnE is secreted and oxidizes sterols from cell walls to generate H_2_O_2_, which acts as a signaling molecule to regulate H_2_O_2_ homeostasis. Oxidative stress is controlled via the natamycin biosynthesis pathway in *S. gilvosporeus*. The intracellular H_2_O_2_ concentration determines the oxidation state of the H_2_O_2_-sensing transcriptional regulator OxyR. By interacting with another transcriptional regulator SgnR, OxyR directly binds to the promoter region of the natamycin biosynthesis pathway-specific transcriptional regulator gene *sgnM* to regulate the expression of genes including *sgnR*, *sgnK*, *sgnS2*, *sgnI*, *sgnJ*, *sgnA*, *sgnE*, *sgnS1*, *sgnD*, *sgnT*, and *sgnH* in the natamycin biosynthesis gene cluster. SgnR–OxyR complexes by cooperation control natamycin biosynthesis by masking or unmasking the −35 region of the *sgnM* promoter depending on the oxidation state of OxyR (reduced, oxidized, or overoxidized), which responds to the intracellular H_2_O_2_ concentration. On the basis of these findings, we propose a dynamic regulation mechanism of natamycin biosynthesis by *Streptomyces* in response to oxidative stress (Fig. 8). This work provides a novel perspective on the crosstalk between intracellular ROS homeostasis and natamycin biosynthesis. Application of these findings will improve antibiotic yields via control of the intracellular redox pressure in *Streptomyces*.

## SUPPLEMENTAL MATERIAL

Supplemental figures and tables spectrum.00879-23-s0001.docx Supplemental figures and tables

Video S1 spectrum.00879-23-s0002.mp4 Conformational changes in OxyR from reduction to oxidation

Video S2 spectrum.00879-23-s0003.mp4 Conformational change between the oxidized and overoxidized states of OxyR

ASM does not own the copyrights to Supplemental Material that may be linked to, or accessed through, an article. The authors have granted ASM a non-exclusive, world-wide license to publish the Supplemental Material files. Please contact the corresponding author directly for reuse.
