# Supplementary material for: Novel mechanism of hydrogen peroxide for promoting efficient natamycin synthesis in Streptomyces
Source: Microbiol Spectr. 2023 Sep 11;11(5):e00879-23. doi: 10.1128/spectrum.00879-23 (PMC10580950; doi:10.1128/spectrum.00879-23)
Supplement: Supplemental figures and tables [file spectrum.00879-23-s0001.docx]

Supplementary Materials for

Novel mechanism of hydrogen peroxide for promoting efficient natamycin synthesis in *Streptomyces*

Gongli Zong^a, b^, Guangxiang Cao^b^, Jiafang Fu^b^, Peipei Zhang^b^, Xi Chen^b^, Wenxiu Yan^b^, Lulu Xin^b^, Zhongxue Wang^b^, Yan Xu^a^, Rongzhen Zhang ^a,^ ^[[1]](#footnote-1)^*

^a^ Key Laboratory of Industrial Biotechnology of Ministry of Education & School of Biotechnology, Jiangnan University, Wuxi 214122, China.

^b^ Biomedical Sciences College & Shandong Medicinal Biotechnology Centre, Shandong First Medical University & Shandong Academy of Medical Sciences, Ji’nan 250117, China

*Corresponding author. Email: rzzhang@jiangnan.edu.cn

**This PDF file includes:**

Supplementary Text

Figures. S1 to S9

Tables S1 to S5

Supplementary Text

Strains and plasmids

General cloning vector pMD18-T and gene expression vector pET-15b were purchased from Takara Biomedical Technology (Beijing) Co., Ltd.(Beijing,China). pJTU1278 and pMS82 were stored by our laboratory ^1, 2^. Strain *S. gilvosporeus* swjs-801 was gift of M. Wang ^3^ (Shandong Academy of Pharmaceutical Science, Shandong, China). The integrity of all expression plasmids was verified by DNA sequencing.

HPLC analysis of natamycin production

Quantitative determination of natamycin was performed by HPLC analysis as described ^3, 4^. Briefly, the culture broth (1 ml) was ultrasonically extracted for 20 min using 9 ml of absolute methanol and then centrifuged at 10,000 ×g for 10 min. HPLC system (Agilent Series 1200, Agilent Technologies, USA) equipped with a Zorbax Eclipse XDB-C18 column was used to determine the natamycin concentration . The mobile phase with a flow rate of 1.0 mL/min, contained ammonium salt solution (0.3 % NH_4_Cl, 0.02 % NH_4_Ac), acetonitrile and tetrahydrofuran in the ratio 75:20:5. An authentic natamycin standard (Sigma, USA) was used to construct a calibration curve of natamycin by HPLC analysis.

EMSA reactions

DNA probes were amplified using primer pairs (Table S4). Labelled DNA probes were then amplified by using EMSABiotin-For and EMSABiotin-Rev (see Table S4 for primer sequences) for 35 PCR cycles. The His6-OxyR protein was incubated with 1 μg of poly(dIdC) (Thermo Scientific, Waltham, MA, USA), 20 mM Tris (pH7.5), 60 mM KCl, 2 mM EDTA, 0.5 mM DTT, 4% Ficoll, and labeled oligonucleotide DNA probes in a total volume of 20 μL. The binding reaction was conducted at room temperature (15-25 ℃) for 30 min. Binding complexes were resolved by electrophoresis on 5% nondenaturing polyacrylamide gels in 0.5 × Tris borate-EDTA buffer at 100 V for 1.5 h at room temperature. For supershift assays, we used ECL chemical luminous agent (GlpBio, Montclair, CA, USA)


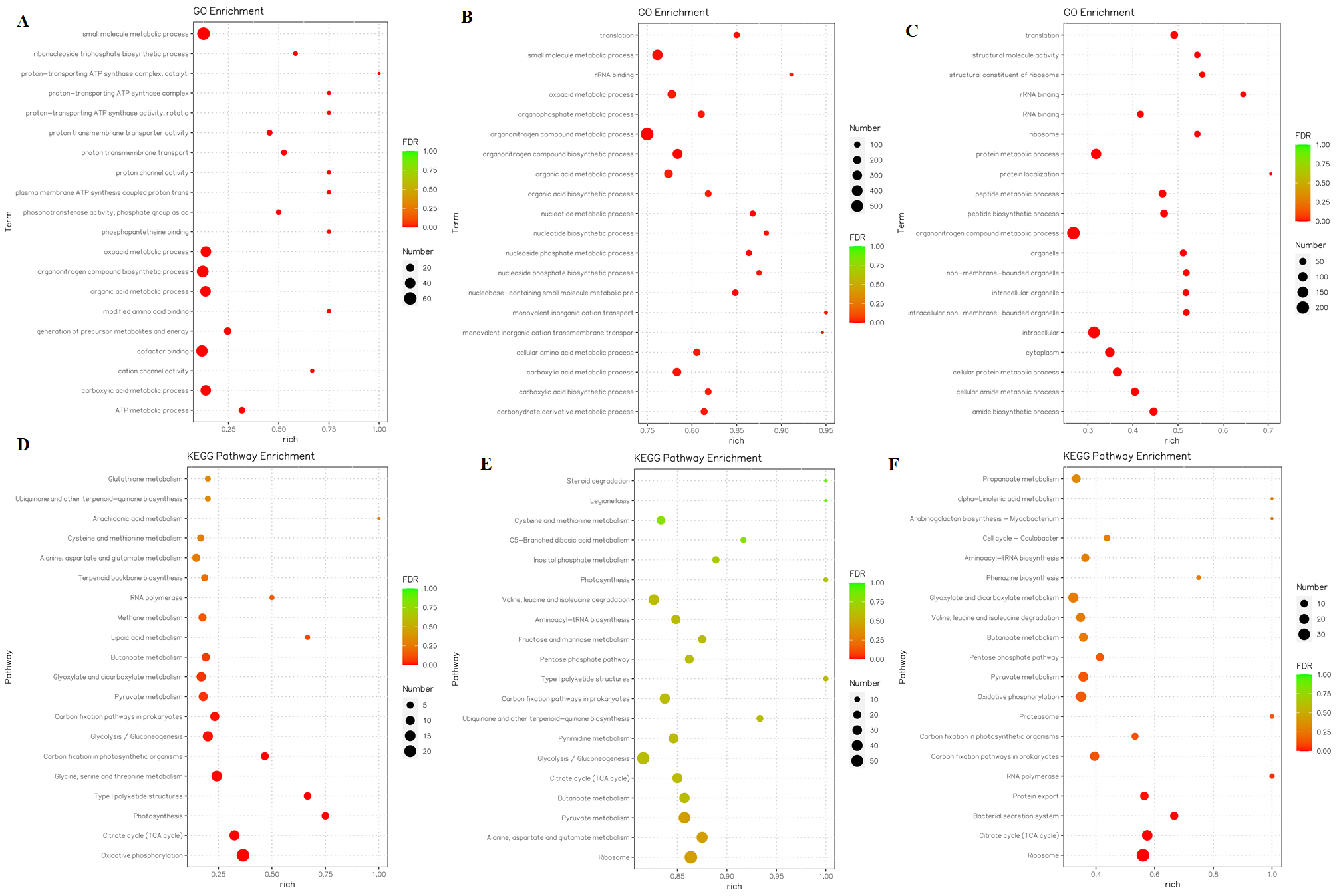


**FIG S1** GO and KEGG rich factors of differentially expressed genes in TC group. (A-C) GO enrichment of differentially expressed genes of *S. gilvosporeus* F607 in TC group compared with TH at 24 h, 60 h and 120 h respectively. (D-F) KEGG pathway GO enrichment of differentially expressed genes of *S. gilvosporeus* F607 in TC group compared with TH at 24 h, 60 h and 120 h respectively.


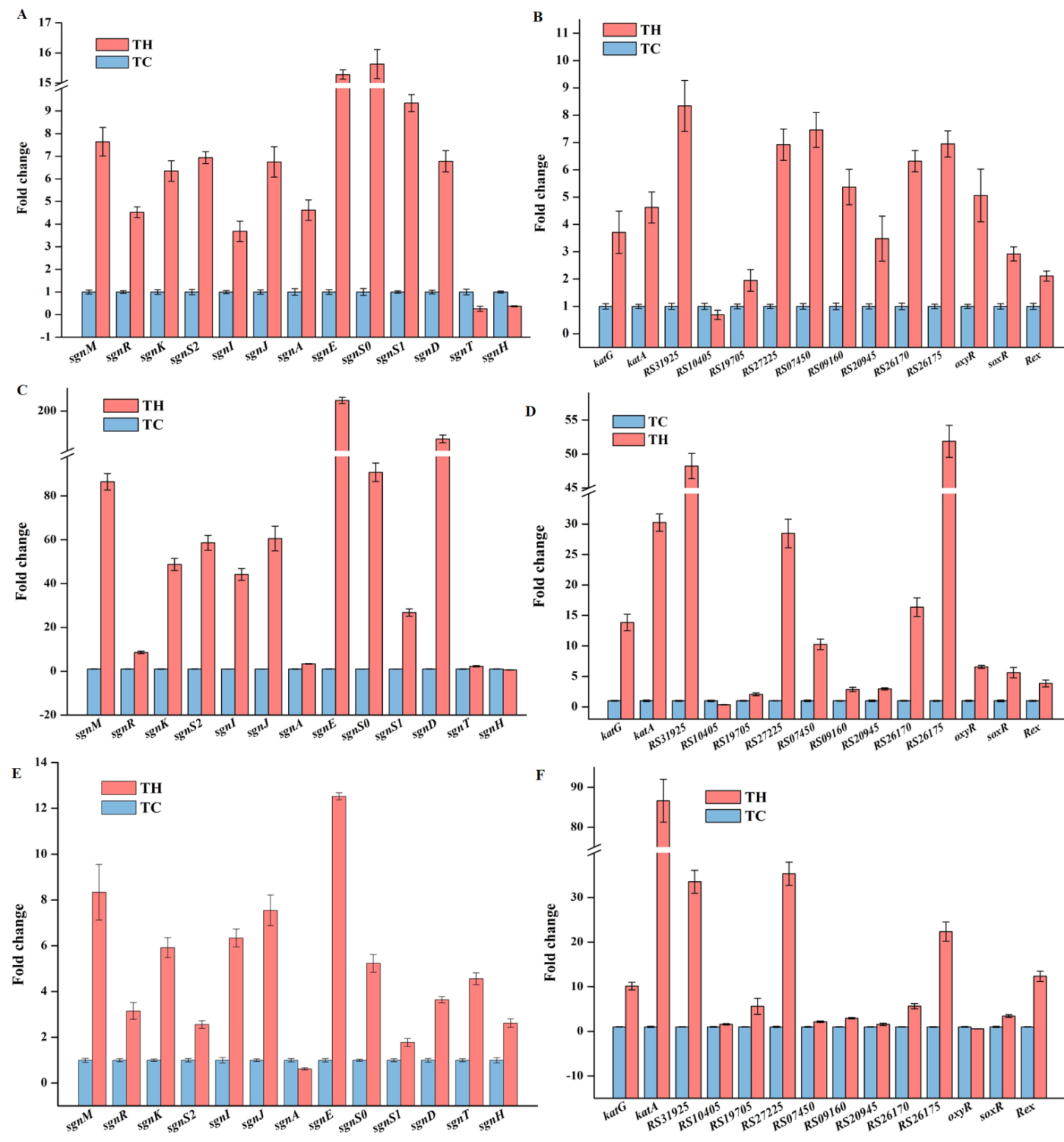


**FIG S2** Transcriptome results by RT-qPCR analyses of some representative genes closely related to natamycin biosynthesis pathway. Transcript levels of natamycin biosynthesis genes and antioxidant-related genes at 24 h (A-B), 60 h (C–D) and 120 h (E–F) of culture. Left, natamycin biosynthesis genes; Right, antioxidant-related genes.

**
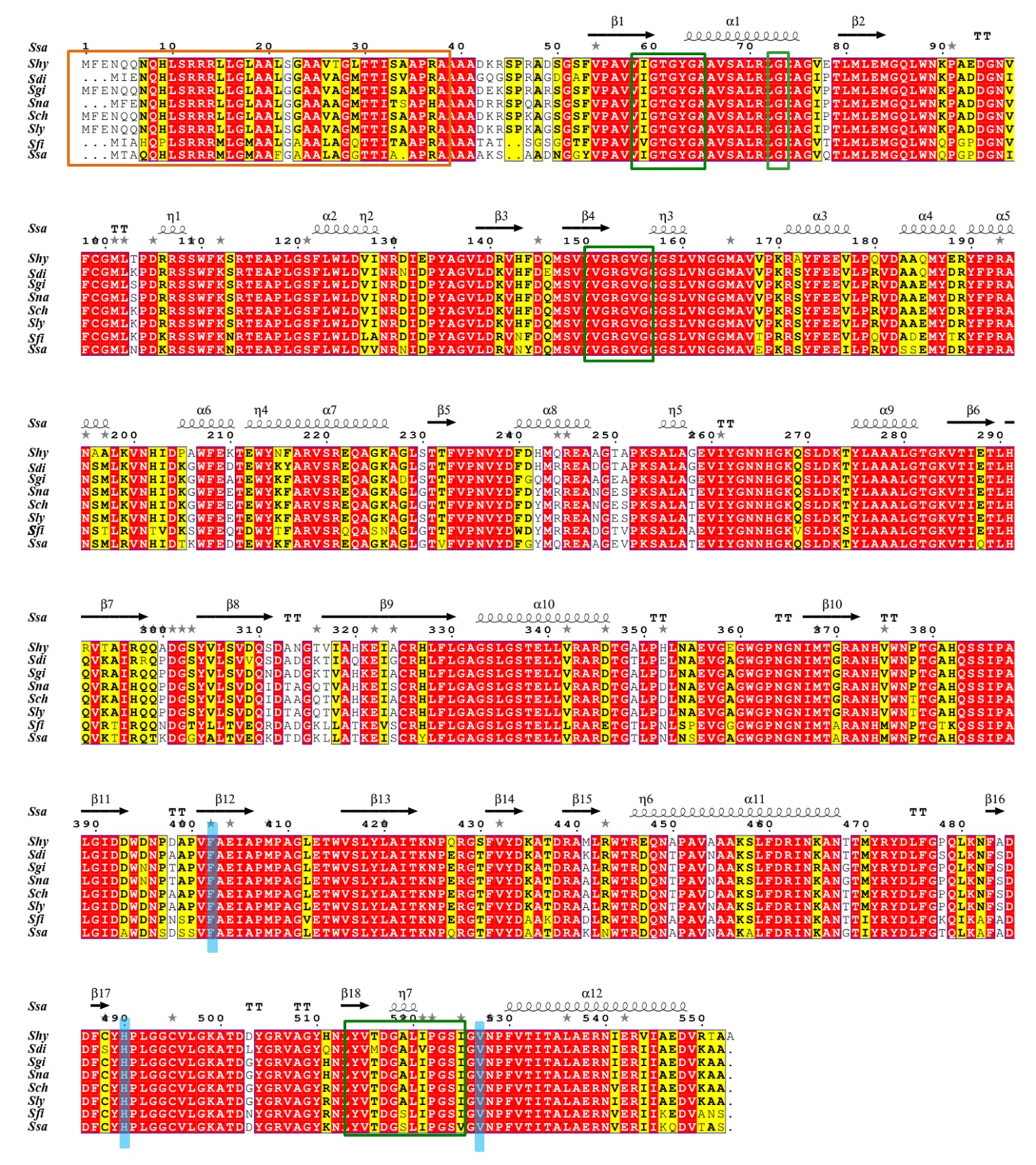
**

FIG S3 Multiple sequence comparison of cholesterol oxidase proteins. Sgi, *Streptomyces gilvosporeus* F607; Eco, *Escherichia coli*; Mle, *Mycobacterium leprae*; Xca, *Xanthomonas campestris*; Vvu1 and Vvu2, Vibrio vulnificus; Sna, *Streptomyces natalensis*; Sly, Streptomyces lydicus; Scn, Streptomyces chattanoogensis; Sco, Streptomyces coelicolor A3(2) Type or paste caption here. Create a page break and paste in the Figure above the caption.


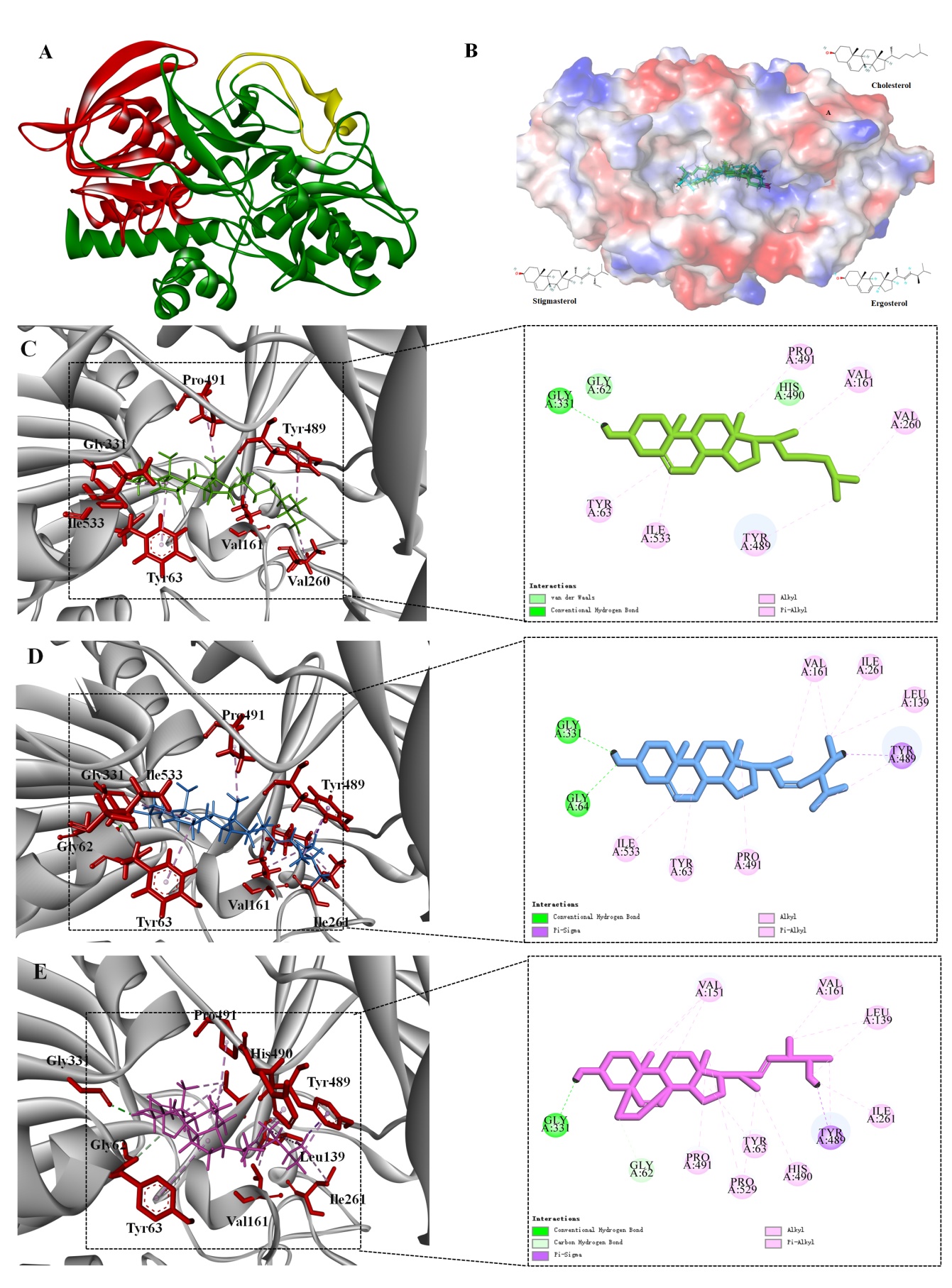
 **FIG S4** Amino acids of SgnE that interact with sterols. (A) Homologous model of SgnE. Red, DNA-binding domain; green, regulatory domain; yellow, cap of substrate-binding domain. Gridlines indicate enzyme active site cavity. (B) Structures of cholesterol, ergosterol, and stigmasterol. (C-E) Interacting amino acids between SgnE and sterols. Left: 3D maps of interacting amino acids between SgnE and sterols. Right: 2D maps of interacting amino acids between SgnE and sterols. Cholesterol, ergosterol, and stigmasterol are shown in blue, carmine, and green, respectively.

**
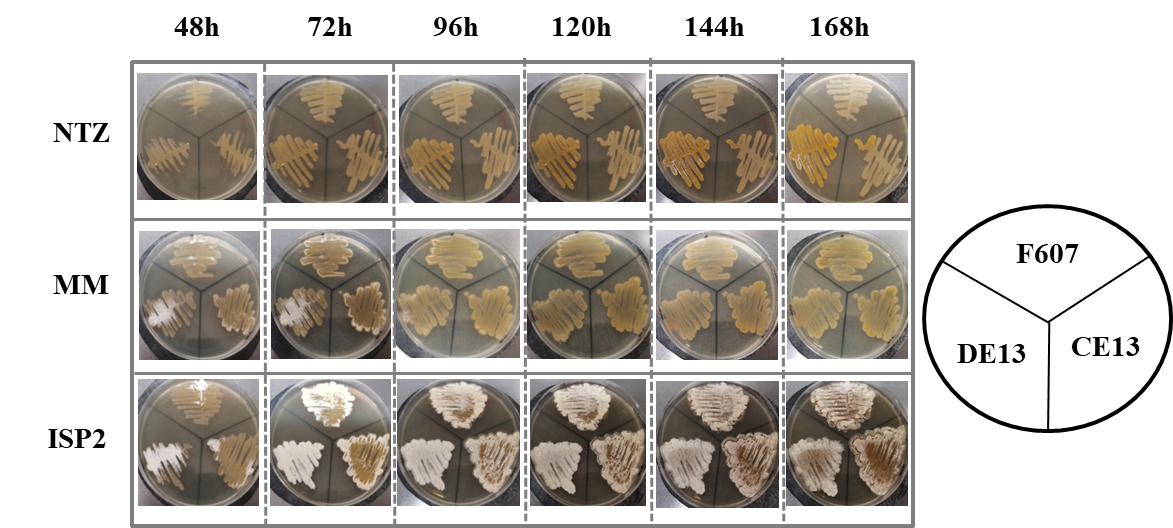
**

**FIG S5** Influence of *sgnE* deletion on spore formation on different solid media.


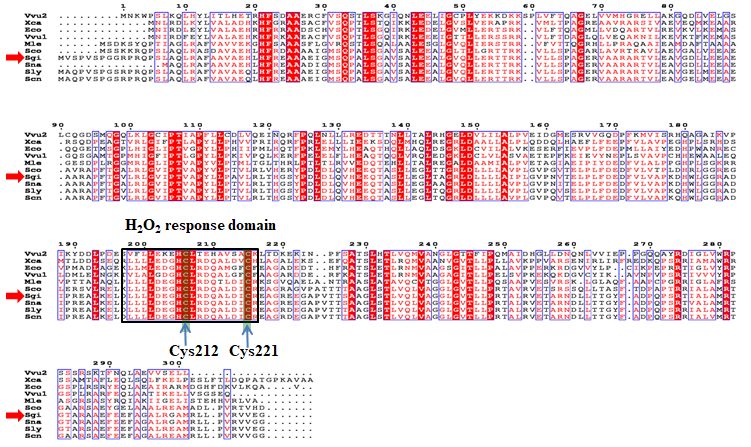


FIG S6 Multiple sequence comparison of OxyR proteins. Sgi, *Streptomyces gilvosporeus* F607; Eco, *Escherichia coli*; Mle, *Mycobacterium leprae*; Xca, *Xanthomonas campestris*; Vvu1 and Vvu2, *Vibrio vulnificus*; Sna, *Streptomyces natalensis*; Sly, *Streptomyces lydicus*; Scn, *Streptomyces chattanoogensis*; Sco, *Streptomyces coelicolor* A3(2) .


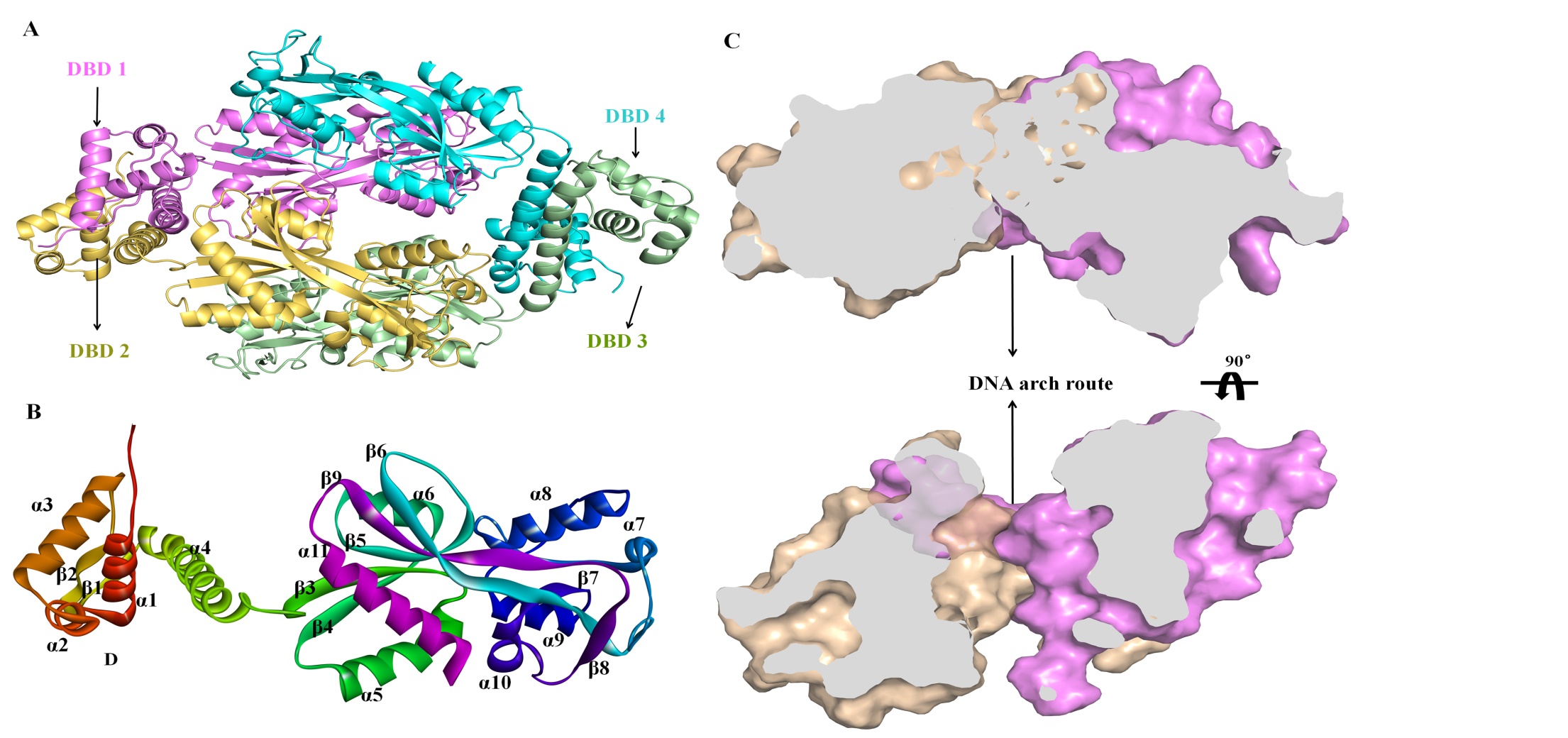


FIG S7 Structural characteristics of OxyR from F607. (A) Tetrameric structure of reduced OxyR from S. gilvosporeus. Left: Each monomer is represented by different colors. Right: Enlarged view of DNA binding domain. (B) OxyR monomer was composed by 11 α-helixes and 9 β-sheets. (C) Cross section of the DNA binding domain and its arch route for the DNA traverse.


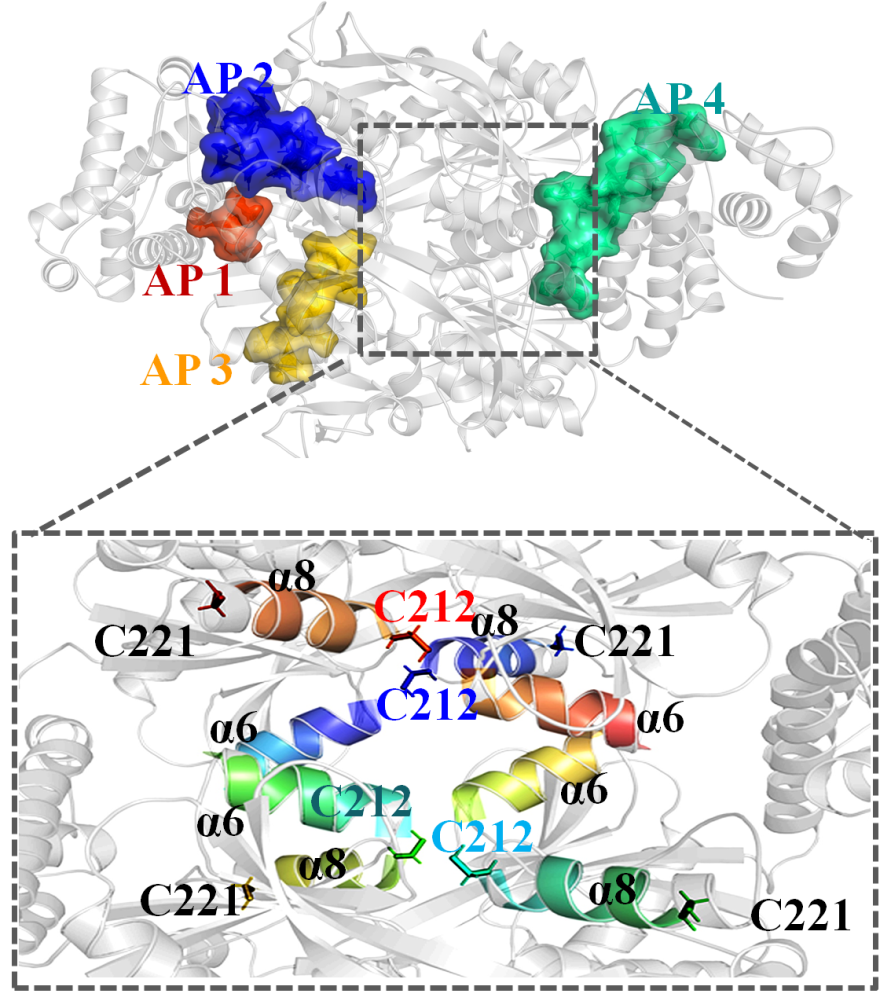


FIG S8. Allosteric sites (bubbles) and H_2_O_2_ reaction pocket of OxyR. The frame of this H_2_O_2_ reaction pocket was formed by eight α-helixes (four α6 and four α8)


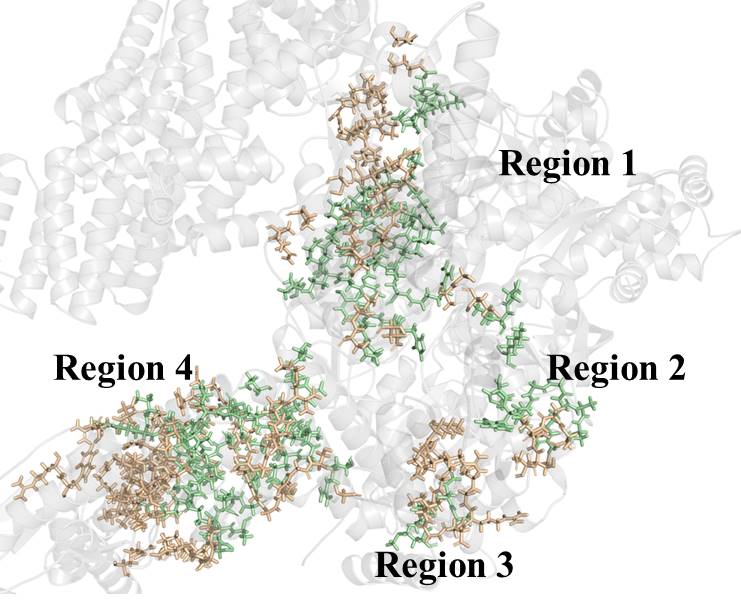


**FIG S9** Four combined regions when the OxyR and SgnR interaction. Residues of OxyR are presented as brown sticks, residues of SgnR are presented as blue-green sticks.

Video S1 : Conformational changes in OxyR from reduction to oxidation

Video S2 : Conformational change between the oxidized and overoxidized states of OxyR

**TABLE S1** Strains and plasmids used in this study

| **Strains and plasmids** | Description |
| --- | --- |
| *S. gilvosporeus* F607 | A high natamycin producing strain |
| *S. gilvosporeus* DE13 | *sgnE* deletion based on *S. gilvosporeus* F607 |
| *S. gilvosporeus* CE13 | *S. gilvosporeus* DE13 complemented with *sgnE* (fusioned with *p_ermE_*) |
| *S. gilvosporeus* swjs-801 | Cholesterol oxidase overexpression strain |
| *S. gilvosporeus* DO7 | *oxyR* deletion based on *S. gilvosporeus* F607 |
| *S. gilvosporeus* CO7 | *S. gilvosporeus* DO7 complemented with *oxyR* (fusioned with *p_ermE_*) |
| *E. coli* DH5α (DE3) | Strain used for gene clone |
| *E. coli* BL21(DE3) | Strain used for protein expression |
| *E. coli* ET12567 (pUZ8002) | Non-methylating donor for conjugation between *E.coli* and *Streptomyces* |
| **Plasmids** |  |
| pMD18-T | General cloning vector |
| pET-15b | Plasmid for *sgnE* and *oxyR* gene expression in *E. coli* |
| pJTU1278 | Plasmid with resistant gene *tsr,* *bla* and oriT for conjugation from *E. coli* to *Streptomyces*. |
| pMS82 | *Streptomyces* integrative vector with hygromycin resistance |

TABLE S2 Primers used in this study

| Primers | Sequences (5’ →3’) | | | | |
| --- | --- | --- | --- | --- | --- |
| **For *sgnE* mutation** | | | | | |
| *sgnE* R-F | | *AACCAGCATC*CAACCCCTTCGTGACCATC | | | |
| *sgnE* R-R | | gcggtggcggccgctctagaTACGCCCAGGTTTCCGACG | | | |
| *sgnE* L-F | | ggggatccactagttctagaGGCGTTCTCCGTGCTGACC | | | |
| *sgnE* L-R | | *CGAAGGGGTTG*GATGCTGGTTCTGCTGGTTCTC | | | |
| *sgnE* V- F1 | | CGGTTCGACAACGTCGGTTTC | | | |
| *sgnE* V- R1 | | ACCGCAGTCGACGAACACGG | | | |
| *sgnE* V- F2 | | GTCATCGTCAACGCCTGAC | | | |
| *sgnE* V- R2 | | CTCCGGGCTCGCAGCAG | | | |
| **For *sgnE* complementation** | | | | | |
| PermE-F1 | | | aagcttGACGTCCATGCGAGTGTCC | | |
| PermE-R1 | | | GGTTCTCGAACGGTTCTGCTGGTTCT | | |
| *sgnE*-Com-F | | | AGAACCAGCAGAACCGTTCGAGAACC | | |
| *sgnE*-Com-R | | | tctagaCTCGAGCTACGCGGCCTTGACGTCCT | | |
| **For SgnE expression** | | | | | |
| *sgnE*-His-F | | | | *catatg*TTCGAGAACCAGCAGAACC (*NdeI*) | |
| *sgnE*-His-R | | | | *ctcgag*CTCGAGCTACGCGGCCTTGACGTCCT (*XhoI*) | |
| **For *oxyR* mutation** | | | | | |
| *oxyR* R-F | | | | TTGATCGGAGGGGGAGCCATGCGGTTGTTGC | |
| *oxyR* R-R | | | | *GCATGCCTGCAGGTCGACGATA*aagcttCTGCTGCTGCCGCTGTCGTG | |
| *oxyR* L-F | | | | *ACCCGGGGATCCTCTAGAGATT*tctagaTCACCAACACGGTCGTCGGT | |
| *oxyR* L-R | | | | CATGGCTCCCCCTCCGATCAACATCGACCA | |
| *oxyR* V- F1 | | | | AGGTGCGTTCACTCGCCGG | |
| *oxyR* V- R1 | | | | ACTGGTCCTGGCACTGGGCT | |
| *oxyR* V- F2 | | | | GTCATCGTCAACGCCTGAC | |
| *oxyR* V- R2 | | | | CTCCGGGCTCGCAGCAG | |
| **For *oxyR* complementation** | | | | | |
| PermE-F2 | | | | tctagaGACGTCCATGCGAGTGTCC | |
| PermE-R2 | | | | CGGTGACACCACTGCTGGTTCTC | |
| *oxyR*-Com-F | | | | GAGAACCAGCAGTGGTGTCACCG | |
| *oxyR*-Com-R | | | | tctagaCTAACCCTCCACCACCCGC | |
| **For OxyR expression and point mutation** | | | | | |
| *oxyR* -His-F | | | | *catatg*GTGGTGTCACCGGTAAGCCC (*NdeI*) | |
| *oxyR* -His-R | | | | *ctcgag*CTAACCCTCCACCACCCGC (*XhoI*) | |
| C212D-L-F | | | | *catatgGCATGCCTGCAGGTCGACGATA*GTGGTGTCACCGGTAAGCCC (*NdeI*) | |
| C212D-L-R | | | | TCGCGCAGGTCGTGGCC | |
| C212D-R-F | | | | GGGCCACGACCTGCGCGAC | |
| C212D-R-R | | | | *ctcgagACCCGGGGATCCTCTAGAGATT*CTAACCCTCCACCACCCGCACCG (*XhoI*) | |
| **For EMSA analysis** | | | | | |
| EMSABiotin-For | | | | *TCTTTCCCTACACGAC | |
| EMSABiotin-Rev | | | | *GTGACTGGAGTTCCTT | |
| p*_sgnMR_*_-1_-F | | | | TCTTTCCCTACACGACgcacaggcccggagctgaag | |
| p*_sgnMR_*_-1_-R | | | | GTGACTGGAGTTCCTTggtcaatgacgcccctgctg | |
| p*_sgnMR_*_-2_-F | | | | TCTTTCCCTACACGACgcaggggcgtcattgacc | |
| p*_sgnMR_*_-2_-R | | | | GTGACTGGAGTTCCTTgtgttcggcaaggattccgac | |
| p*_sgnMR_*_-3_-F | | | | TCTTTCCCTACACGACctgccgctttcttgccattc | |
| p*_sgnMR_*_-3_-R | | | | GTGACTGGAGTTCCTTtgcggtccccgtggttctc | |
| p*_sgnMR_*_-2_ | | | | *ACCGCCCTTGCGG**GGTCC**TGGTCGAGCCACTGG**CGGC**TCCTTGCCGTCGCCT**GCCG**TCGTGTTCC**GGACC**CCTTGTCGGAATC | |
| p*_sgnMR_*_-2_-M1 | | | | *ACCGCCCTTGCGG**GGTCC**TGGTCGAGCCACTGG**TACT**TCCTTGCCGTCGCCT**GCCG**TCGTGTTCC**GGACC**CCTTGTCGGAATC | Motif 2 mutation |
| p*_sgnMR_*_-2_-M2 | | | | *ACCGCCCTTGCGG**GGTCC**TGGTCGAGCCACTGG**TACT**TCCTTGCCGTCGCCT**CAGC**TCGTGTTCC**GGACC**CCTTGTCGGAATC | Motif 2 and 3 mutation |
| p*_sgnMR_*_-2_-M3 | | | | *ACCGCCCTTGCGG**ATGTT**TGGTCGAGCCACTGG**CGGC**TCCTTGCCGTCGCCT**GCCG**TCGTGTTCC**GGACC**CCTTGTCGGAATC | Motif 4 mutation |
| p*_sgnMR_*_-2_*-*M4 | | | | *ACCGCCCTTGCGG**ATGTT**TGGTCGAGCCACTGG**CGGC**TCCTTGCCGTCGCCT**GCCG**TCGTGTTCC**TCGCA**CCTTGTCGGAATC | Motif 1 and motif 4 mutation |
| p*_sgnMR_*_-2_*-*M5 | | | | *ACCGCCCTTGCGG**ATGTT**TGGTCGAGCCACTGG**TCAT**TCCTTGCCGTCGCCT**GCCG**TCGTGTTCC**GGACC**CCTTGTCGGAATC | Motif 3 and motif 4 mutation |
| p*_sgnMR_*_-2_-M6 | | | | *ACCGCCCTTGCGG**ATGTT**TGGTCGAGCCACTGG**TCAT**TCCTTGCCGTCGCCT**CAAG**TCGTGTTCC**TCGCA**CCTTGTCGGAATC | Motif 1,2,3 and 4 mutation |
| p*_sgnMR_*_-2_-M7 | | | | *ACCGCCCTTGCGG**ATGTT**TGGTCGAGCCACTGG**TCAT**TCCTTGCCGTCGCCT**CAAG**TCGTGTTCC**GGACC**CCTTGTCGGAATC | Motif 1, 2 and 3 mutation |

| TABLE S3 Primers for Real-time PCR analysis used in this study | | |
| --- | --- | --- |
| *sgnM* RT F | AAGCACCAGCGATTCGTCAC |  |
| *sgnM* RT R | ATCGCGGAGAGGATCTTCTTG |  |
| *sgnR* RT F | CACCTCCGACCGCAAGCG |  |
| *sgnR* RT R | TGAGGAGCAGGGCGAGCAG |  |
| *SgnS0* RT F | ACGGGAACTGGGCGGCTACAC |  |
| *SgnS0* RT R | GATACCCGACTCCTGCAACCCG |  |
| *SgnS1* RT F | TTCGTTGTCGAGCAGGTCG |  |
| *SgnS1* RT R | CTTCGGCGGACTCGACATC |  |
| *sgnS2* RT F | GCGACCGGACTGAACCTGAC |  |
| *sgnS2* RT R | CTTCAGCAGGGGTTCGAGCAC |  |
| *sgnK* RT F | GCTCAATCCGCTGCTCGTGC |  |
| *sgnK* RT R | GGCGAACTCCACAGGGG |  |
| *sgnI* RT F | GAGGTGTTCACGCAGAAGTGCC |  |
| *sgnI* RT R | TGACGACGACCCCAAGACG |  |
| *sgnJ* RT F | GTCGATGCTGACGTAGTCCTC |  |
| *sgnJ* RT R | TGTCCCTGGGCAACCTC |  |
| *sgnA* RT F | CGACAGCGGCTATATCACCC |  |
| *sgnA* RT R | CACATCGTTGACGGTCCGC |  |
| *sgnD* RT F | AAGATGCTGGAGCTGAGCCC |  |
| *sgnD* RT R | ATCAGCAGGTCCAGGAAGGG |  |
| *sgnT* RT F | GATCGGCCTGGGCGTGAC |  |
| *sgnT* RT R | GTCCCCCTCCAGATGATGGTG |  |
| *sgnH* RT F | AGGAACGTGCCGTAGCCG |  |
| *sgnH* RT R | GTCGCCTTCGGCTACCTCTC |  |

**TABLE S4** Differential expression of genes associated with natamycin biosynthesis and antioxidant stress system.

| Gene ID | Gene | Product | Function | Fold change of transcriptome (TH/TC) | | |
| --- | --- | --- | --- | --- | --- | --- |
|  |  |  |  | TC24_vs_TH24 | TC60_vs_TH60 | TC120_vs_TH120 |
| genes associated with natamycin biosynthesis | | | |  |  |  |
| gene-B1H19_RS05005 | *sgnM* | PAS-LuxR transcriptional regulator | Transcriptional regulation | 6.90 | 100.48 | 7.29 |
| gene-B1H19_RS05010 | *sgnR* | SARP-LAL transcriptional regulator | Transcriptional regulation | 2.86 | 6.36 | 3.50 |
| gene-B1H19_RS05015 | *sgnK* | MGT family glycosyltransferase | Mycosamine transferase | 5.81 | 53.26 | 5.38 |
| gene-B1H19_RS05020 | *sgnS4* | Polyketide synthase | PKS Extension module 12, cyclisation and chain release | 16.72 | 157.76 | 3.02 |
| gene-B1H19_RS05025 | *sgnS3* | Polyketide synthase | PKS Extension module 11 | 13.04 | 68.33 | 1.82 |
| gene-B1H19_RS05030 | *sgnS2* | Polyketide synthase | PKS Extension modules 5-10 | 7.99 | 83.35 | 2.12 |
| gene-B1H19_RS05035 | *sgnI* | Thioesterase | Thioesterase | 5.58 | 45.15 | 5.41 |
| gene-B1H19_RS05040 | *sgnJ* | GDP-mannose4,6-dehydratase | GDP-mannose4,6-dehydratase | 7.82 | 74.16 | 7.81 |
| gene-B1H19_RS05045 | *sgnA* | ABC transporter | Natamycin export | 2.12 | 2.65 | 0.85 |
| gene-B1H19_RS05050 | *sgnB* | ABC transporter | Natamycin export | 1.57 | 2.112 | 0.69 |
| gene-B1H19_RS05055 | *sgnE* | Cholesterol oxidase | / | 10.34 | 335.10 | 15.58 |
| gene-B1H19_RS05060 | *sgnC* | Aminotransferase | GDP-3-keto-6-deoxymannose aminotransferase | 10.27 | 394.60 | 11.98 |
| gene-B1H19_RS05065 | *sgnG* | Cytochrome P450 | C12 hydroxylase | 6.71 | 204.94 | 5.37 |
| gene-B1H19_RS05070 | *sgnF* | Ferredoxin | Ferredoxin | 5.07 | 50.297 | 1.74 |
| gene-B1H19_RS05075 | *sgnS0* | Polyketide synthase | PKS Loading module | 12.23 | 129.47 | 4.67 |
| gene-B1H19_RS05080 | *sgnL* | Tyrosine phosphatase | Tyrosine phosphatase | 5.99 | 3.388 | 0.54 |
| gene-B1H19_RS05085 | *sgnS1* | Polyketide synthase | PKS Extension modules 1-4 | 6.09 | 25.40 | 1.54 |
| gene-B1H19_RS05090 | *sgnD* | cytochrome P450 | C4,5 epoxidase | 8.32 | 175.30 | 3.30 |
| gene-B1H19_RS05120 | *sgnT* | LysE family translocator | Natamycin inducer PI factor secretion | 0.59 | 1.53 | 2.11 |
| gene-B1H19_RS05145 | *sgnH* | MFS transporter (Macrolide efflux protein A) | PIM efflux pump | 0.11 | 0.611 | 1.93 |
| gene-B1H19_RS05150 | Un | Decarboxylase | / | 0.16 | 0.98 | 3.31 |
| gene-B1H19_RS05155 | Un | Iron transporter | / | 0.12 | 0.92 | 3.97 |
| **genes associated with antioxidant stress system** | | | | | | |
| gene-B1H19_RS01770 | *katG* | Catalase |  | 2.42 | 12.02 | 8.98 |
| gene-B1H19_RS26930 | *katA* | Catalase | H_2_O_2_ decomposition | 3.41 | 36.11 | 97.63 |
| gene-B1H19_RS31925 | Un | Catalase | H_2_O_2_ decomposition | 6.13 | 61.03 | 26.57 |
| gene-B1H19_RS10405 | Un | NAD(P)H dehydrogenase | Electron reduction | 1.74 | 0.34 | 1.50 |
| gene-B1H19_RS19705 | Un | GRX glutaredoxin redoxin | glutathione S-transferase | 2.27 | 1.74 | 5.20 |
| gene-B1H19_RS27225 | Un | Superoxide dismutase | Antioxidant enzyme | 6.07 | 30.36 | 40.20 |
| gene-B1H19_RS07450 | Un | Glutathione peroxidase | Antioxidant enzyme | 5.73 | 10.69 | 1.77 |
| gene-B1H19_RS09160 | Un | Carboxymuconolactone decarboxylase | Antioxidant enzyme | 3.58 | 2.614 | 3.04 |
| gene-B1H19_RS20945 | Un | Alkyl hydroperoxide reductase | Thiol-specific antioxidant | 1.51 | 2.84 | 1.04 |
| gene-B1H19_RS26170 | Un | Alkyl hydroperoxide reductase | Thiol-specific antioxidant | 6.73 | 18.08 | 5.78 |
| gene-B1H19_RS26175 | Un | Alkyl hydroperoxide reductase | Thiol-specific antioxidant | 7.38 | 85.79 | 24.90 |
| gene-B1H19_RS26180 | *oxyR* | Redox-sensing transcriptional regulator OxyR | Transcriptional regulation in antioxidant stress system | 2.54 | 1.34 | 0.80 |
| gene-B1H19_RS10530 | *soxR* | Redox-sensing transcriptional regulator SoxR | Transcriptional regulation in antioxidant stress system | 2.58 | 5.04 | 3.62 |
| gene-B1H19_RS19700 | *Rex* | Redox-sensing transcriptional regulator Rex | Transcriptional regulation in antioxidant stress system | 1.56 | 3.69 | 11.36 |

Un: unnamed genes; /: unclarified function

TABLE S5 Allosteric sites prediction of OxyR

|  | Pocket Volume | Total SASA | Feature Score | Perturbation Score | AlloSite Score |
| --- | --- | --- | --- | --- | --- |
| Pocket1 | 2571.94 | 1513.606 | 0.470 | 1.000 | 0.576 |
| Pocket2 | 3469.854 | 1656.046 | 0.399 | 1.000 | 0.519 |
| Pocket3 | 1957.443 | 976.655 | 0.414 | 1.000 | 0.531 |
| Pocket4 | 763.011 | 338.664 | 0.413 | 1.000 | 0.530 |

1. He, Y., Wang, Z., Bai, L., Liang, J., Zhou, X. & Deng, Z. Two pHZ1358-derivative vectors for efficient gene knockout in *Streptomyces*. *J Microbiol Biotechnol* **20**, 678-682 (2010).

2. Gregory, M. A., Till, R. & Smith, M. C. Integration site for *Streptomyces* phage phiBT1 and development of site-specific integrating vectors. *J Bacteriol* **185**, 5320-5323 (2003).

3. Wang, M. et al. Improvement of natamycin production by cholesterol oxidase overexpression in *Streptomyces gilvosporeus*. *J Microbiol Biotechnol* **26**, 241-247 (2016).

4. Enriquez, L. L. et al. An efficient gene transfer system for the pimaricin producer Streptomyces natalensis. *FEMS Microbiol Lett* **257**, 312-318 (2006).

1. * Corresponding author at: Key Laboratory of Industrial Biotechnology of Ministry of Education & School of Biotechnology, Jiangnan University, Wuxi 214122, P. R. China.

   *E-mail addresses:* rzzhang@jiangnan.edu.cn [↑](#footnote-ref-1)
